# A bifunctional fusion membrane-based biocompatible nanovaccine to potentiate cancer immunotherapy

**DOI:** 10.7150/thno.106376

**Published:** 2025-04-21

**Authors:** Wei Fu, Xing Cai, Jinru Yang, Lian Yang, Yaoyu Pan, Zhan Tuo

**Affiliations:** 1 Department of Oncology, The First Affiliated Hospital of Chongqing Medical University, Chongqing 400016, China.; 2 Department of Radiation and Medical Oncology, Zhongnan Hospital of Wuhan University, Wuhan 430071, China.; 3 Department of Radiology, Union Hospital, Tongji Medical College, Huazhong University of Science and Technology, Wuhan, 430022, China.; 4 Hubei Key Laboratory of Molecular Imaging, Wuhan, 430022, China.; 5 Department of Polymer, School of Material Science and Engineering, Hubei University, Wuhan, Hubei 430062, China.; 6 The Affiliated Cancer Hospital of Zhengzhou University & Henan Cancer Hospital, Zhengzhou 450008, China.

**Keywords:** biomimetic nanovaccine, senescent tumor cell, escherichia coli, immunotherapy, postoperative recurrence

## Abstract

**Background:** Cancer cell membrane-based nanovaccines derived from patients' tumor tissues have shown promising features as a personalized cancer treatment strategy. However, the weak immunogenicity of autologous tumor antigens undermines the therapeutic effects of personalized vaccines.

**Methods**: We synthesized a biomimetic nanovaccine, Bio-HCP@FM-NPs, composed of senescent tumor cell membranes, *Escherichia coli* cytoplasmic membrane extracts, and granulocyte-macrophage colony-stimulating factor (GM-CSF)-encapsulated biocompatible hypercross-linked polymer nanoparticles. The nanovaccine's antitumor and enhanced immunotherapy effects were demonstrated in multiple tumor models. The tumor prevention effects of nanovaccine were assessed using a postoperative recurrence model.

**Results:** The Bio-FM@HCP-NP vaccine showed promising therapeutic efficacy in the B16-F10 melanoma mouse model and significantly synergized with anti-PD-1 immunotherapy across multiple tumor models. Mechanistically, GM-CSF was promptly released to recruit naïve DCs to the nanovaccine. Thereafter, immature DCs were vigorously activated by FM-NPs, thereby activating the cytotoxic T cells. Furthermore, Bio-HCP@FM-NPs induced robust antigen-specific immune responses, prolonging postoperative survival in mice and providing long-term protection against tumor recurrence. Targeted depletion of immune cell populations revealed that T and B cells were essential for vaccine-induced tumor regression.

**Conclusion:** The Bio-HCP@FM-NPs showed significant promise for immunotherapy and tailored postoperative treatment of cancer, leveraging the strong activation of innate immunity by senescent tumor cell membranes and bacterial cytoplasmic membrane extracts.

## Introduction

The orchestration of the immune system has shown clinical success in treating various lethal malignancies using adaptive immune cell transfer, immune checkpoint inhibitors, and vaccines 1-4. A promising strategy involves the use of antigens derived from patients' tumor tissues to generate personalized cancer vaccines, triggering immune responses and eliminating residual cancer cells 5-8. Personalized tumor vaccines, particularly those based on tumor cell membranes, are enriched with tumor-associated antigens and have been utilized in treating various cancers 9. However, despite their potential, these vaccines have not yet shown long-term therapeutic efficacy. This limitation may stem from their weak immunogenicity, primarily due to insufficient activation of antigen-presenting cells, particularly dendritic cells (DCs) 10, 11. Thus, novel vaccine formulations are urgently required to elicit durable immune responses and achieve clinical efficacy.

Cellular senescence is a major obstacle to solid tumor progression 12-14. The senescence of tumor cells in response to genotoxic agents or other medications can suppress uncontrolled cell proliferation. Moreover, aging tumor cells may acquire a senescence-associated secretory phenotype (SASP) 15, 16. This phenotype relays stress signals to neighboring immune cells. These senescent cells are highly immunogenic. They efficiently transfer antigens, release immunogenic SASP components, and activate antigen-presenting cells (APCs) to trigger robust anti-tumor immune responses 17. Therefore, senescent tumor cells can recruit both innate and adaptive immune cells, thereby outcompeting their non-senescent counterparts in inducing tumor regression. Nevertheless, oncogenic effects may occur with prolonged administration of vaccines derived from senescent tumor cells 18. SASP is a double-edged sword in immune modulation. It can increase the abundance of immunosuppressive myeloid cells and inhibit the anti-tumor responses of T cells and Natural Killer (NK) cells, thereby promoting tumor progression, therapy resistance, and relapse 19, 20. Thus, ensuring efficient antigen presentation while minimizing SASP-mediated disruption of the immune response is critical for the therapeutic efficacy of vaccines derived from senescent tumor cell membranes.

Compounds originating from bacteria can induce immune cells to respond to external "danger signals" via the innate immune system 21-23. In cancer vaccines, bacterial extracts can enhance anti-tumor immune responses by modulating the adaptive immune response 24-27. Some bacterial cancer vaccines have been investigated in clinical trials 28, 29. Bacterial vaccines cannot induce long-term immune memory against tumor recurrence 21. Moreover, bacterial formulations can lead to severe side effects, such as cytokine storm and sepsis, limiting their clinical application 24, 25. The bacterial cytoplasmic membrane is distinct from the cell wall and can be extracted. It does not contain lipopolysaccharides (LPS) and other harmful elements of the cell wall 30. Therefore, the bacterial cell membrane, devoid of LPS and other harmful elements, can serve as a potential adjuvant to ameliorate these danger signals.

Membrane fusion is a technology that can be used to create a fusion membrane (FM) possessing the characteristics of different cell membranes 31-33. Based on this technology, we produced fusion membrane nanoparticles (FM-NPs) by merging E. coli membrane extracts (EMs) with cisplatin-induced autologous senescent tumor cell membranes (STCMs) from excised tumor tissues to introduce tumor antigens and concurrently stimulate dendritic cells (DCs). Subsequently, FM-NPs and granulocyte-macrophage colony-stimulating factor (GM-CSF) were incorporated into uniform core-shell structured biocompatible hypercross-linked polymer nanoparticles (Bio-HCP-NPs). This combination provided a customized Bio-HCP@FM-NPs-based vaccine, leveraging the cytocompatible surface groups and unique internal microporous characteristics of the material. Personalized tumor vaccine was safely employed as an activator of the innate immune system, optimizing anti-tumor effects while minimizing side effects. Moreover, the Bio-HCP@FM-NPs-based vaccine, in conjunction with anti-PD-1, demonstrated strong anti-tumor effects and prolonged the survival of mice with lung cancer (LLC) and melanoma (B16F10) cell models. In this study, we first utilized Bio-HCP-NPs as carriers in the field of biomedical engineering/biotherapy. Compared to traditional carrier materials, such as mesoporous silica, metal-organic frameworks, and amphiphilic block polymers, the Bio-HCP-NPs exhibited controllable size, rich surface functional groups, high surface area, low skeleton density, strong chemical stability, and low biological toxicity 34-36. Taken together, our study revealed that dual-function fusion membranes combined with Bio-HCP-NPs can act as a customized tumor vaccine, enhancing the response to cancer immunotherapy.

## Material and methods

### Materials

Cell Counting Kit-8 (CCK-8 kit) was obtained from Sigma-Aldrich (96992). GM-CSF was purchased from MCE (HY-P7361). Interleukin-4 (IL-4) was purchased from MCE (HY-P70653). Polystyrene and polyethylene were purchased from Sigma-Aldrich (450383, 428043). FeCl_3_ and dimethoxymethane were purchased from Sigma-Aldrich (701122, D134651).

### Synthesis of PS@PEG-NPs

The Polystyrene@polyethylene glycol nanoparticles (PS@PEG-NPs) were synthesized by emulsion polymerization 37. A mixture of sodium hydroxide (1 mg/mL), sodium bicarbonate (1 mg/mL), Sodium dodecyl sulfate (2 mg), and potassium persulfate (30 mg) was stirred in a nitrogen environment for 30 min. Subsequently, styrene (300 µL), ethylene glycol dimethacrylate (30 µL), and divinylbenzene (3 µL) monomers were mixed and added to the above aqueous solution dropwise at a constant rate. The reaction was stirred at 400 rpm at 70 °C for 12 h. After the reaction, excess initiators and monomers were removed by dialysis and centrifugation.

### Synthesis of Bio-HCP-NPs

The biocompatible hypercrosslinked Polystyrene@polyethylene glycol nanoparticles (Bio-HCP-NPs) were synthesized by the Friedel-Crafts Alkylation via direct knitting strategy 38, 39. The previously prepared polystyrene@polyethylene glycol nanoparticles (1 g) were dispersed in 100 mL of 1,2-dichloroethane. While maintaining magnetic stirring and a nitrogen atmosphere, FeCl_3_ catalyst and dimethoxymethane (FDA) were added. The molar ratio of the three chemicals was 1:2:2 (nanoparticles: FeCl_3_: FDA). The reaction was then carried out at 80 °C for 48 h. The final product was centrifuged and washed sequentially with 1,2-DCE, MeOH, and deionized water. Finally, the product was dried by lyophilization.

### Cell culture

Cell lines, mouse-derived Lewis lung carcinoma (LLC) cells and mouse-derived melanoma (B16-F10) cells were obtained from the American Tissue Culture Collection (ATCC). Primary immune cells (Dendritic cells and T cells) from mice and B16-F10 cells were maintained in the RPMI-1640 medium, while LLC cells were maintained in the DMEM medium. Each medium was supplemented with 10% heat-inactivated fetal bovine serum (FBS) from Gibco and 1% antibiotics (100 U/mL penicillin/streptomycin; Gibco). Cells were cultured in an incubator with 5% CO_2_ and maintained humidity at 37 ºC.

### Senescence cell induction

Senescence was induced through incubation with cisplatin at different concentrations (50 nM, 100 nM, 200 nM) for 5 days. Following the treatments, these cells were harvested and used for senescence-associated β-galactosidase staining to verify the success of the induced- senescence.

### Senescence-associated β-galactosidase assay

Cells were washed with PBS three times and fixed with 4% paraformaldehyde. Subsequently, cells were marked using a senescence β-galactosidase staining kit (Beyotime, C0602) following the manufacturer's instructions.

### Preparation of EM-NPs

The freeze-dried *Escherichia coli* (E. coli, CCTCC AB 93154) powder was added to 8 mL of Luria-Bertani (LB) culture. They were incubated at 37 °C with shaking at 200 rpm in a temperature-controlled shaker until the bacterial density reached 1 x 10^8^ CFU/mL. All bacterial samples were washed by centrifugation (4000 rpm, 5 min) and with PBS at least three times. Subsequently, the pellets were collected and repeatedly rinsed with PBS. Before the experiment, bacterial concentrations were measured using the plate counting technique and then diluted to the required concentration. The obtained bacterial pellets were disrupted by an ultrasonic reactor (Sonic Materials Inc., USA) for 25 min (40 W, 2 s). Afterward, the liquid above the solid was separated by centrifugation at 1000 rpm for 15 min. The clear liquid was sterilized in an autoclave for 30 min. Subsequently, it was frozen and freeze-dried to obtain the E. coli membrane extracts (EMs) powder. To dissolve EMs, 50 μL of mineral oil was initially employed to dissolve 5 mg of freeze-dried powder. Thereafter, 36 μL of Tween 80 and 964 μL of double-distilled water were added to achieve a concentration of 5 mg/mL formulations containing EMs. To fabricate E. coli membrane extracts-derived nanoparticles (EM-NPs), the composite mixtures underwent multiple extrusion cycles (≥ 30 passes) through a precision extruder equipped with a 100 nm pore-size membrane. The size distribution for EM-NPs was measured using dynamic light scattering (DLS) with a Brookhaven BI9000AT system from Brookhaven Instruments Co.

### Preparation of TCM-NPs and STCM-NPs

To isolate tumor cell membranes, untreated or senescent B16-F10 and LLC cells were resuspended at 1 × 10⁸ cells/mL and lysed via six freeze-thaw cycles. After centrifugation at 700 g for 10 min, precipitates were removed. Afterward, the clear liquid was subjected to sonication at half power (125 W, 20 kHz) for 2 min and then centrifuged again (14,000 × g, 30 min). The precipitate containing tumor cell membranes (TCMs) and senescent tumor cell membranes (STCMs) were collected and stored. The TCM nanoparticles (TCM-NPs) and STCM nanoparticles (STCM-NPs) were fabricated by extruding the membranes through a 100 nm pore-sized filter (≥ 30 cycles). Hydrodynamic size distribution was quantitatively analyzed using a Brookhaven BI9000AT DLS system (Brookhaven Instruments Corporation, USA).

### Synthesis and analysis of Bio-HCP@FM-NPs

To prepare hybrid cell membrane nanoparticles (FM-NPs), EMs-NPs (100 μg) and STCM-NPs (50 μg) were sonicated at 50% amplitude (125 W, 20 kHz, 2 min) and extruded ≥ 30 times through a 100-nm membrane. Bio-HCP@FM-NPs were then fabricated by co-extruding FM-NPs with Bio-HCP-NPs (10 mg/mL) through a 100 nm filter ( ≥ 30 cycles). Size distributions were analyzed via dynamic light scattering (DLS; Brookhaven BI9000AT system, Brookhaven Instruments Corporation).

### DCs generation and activation

Bone marrow cells from *C57BL/6* mice were cultured in RPMI-1640 medium with 20 ng/mL of murine GM-CSF and 10 ng/mL of murine IL-4 to produce bone marrow-derived dendritic cells. Red blood cells (RBCs) were broken down using RBC lysis buffer (Biosharp) for 5 min at room temperature (25 °C). Fresh medium with 20 ng/mL of murine GM-CSF was added to the culture every other day. Bone marrow-derived dendritic cells (BMDCs) were collected on day 7 after induction.

### Evaluation of DC and T cell activation elicited by STCMs *in vitro*

To assess STCM effects on DC maturation and T cell activation, 1 × 10⁵ BMDCs/well in 6-well plates were exposed to PBS, LPS (100 ng/mL), TCMs, EMs, and STCMs (150 µg/mL, each) for 48 h. Cells were then centrifuged (500 × g, 5 min) and re-suspended in FACS Buffer. Then, the other BMDCs pre-treated with nanoparticles were co-cultured 1 × 10^6^/well spleen cells extracted from the spleens of *C57BL/6* mice for 72 h and then collected the cells. The collected cells were re-suspended in FACS Buffer. Subsequently, the T cell activation and DCs maturation were assessed using flow cytometry. Fluorescence labeling was conducted using CD11c (BioLegend, 101228), MHC-II (BioLegend, 107613), MHC-I (BioLegend, 343303), CD80 (BioLegend, 104707), CD86 (BioLegend, 105011), CD45 (BioLegend, 103137; 157214), CD3 (BioLegend, 100236), CD4 (BioLegend, 100509), CD8a (BioLegend, 100722), IFN-γ (BioLegend, 505830) and granzyme B (GrzB, BioLegend, 372208) tagged with fluorescein isothiocyanate. Cells were analyzed using a BD FACSAria™ Fusion cell sorter (BD Biosciences, USA) and CytExpert software.

### Assessment of dual T-Cell activation elicited by Bio-HCP@FM-NPs *in vitro*

To assess the direct T cell immunostimulatory response triggered by Bio-HCP@FM-NPs, we extracted immune cells from the spleens of *C57BL/6* mice. Thereafter, the immune cells were seeded at a concentration of 1 × 10^6^ cells/well in a 6-well plate and co-cultured with PBS, Bio-HCP-NPs, Bio-HCP@EM-NPs, Bio-HCP@STCM-NPs or Bio-HCP@FM-NPs (each 150 μg/mL) for 72 h. Subsequently, the expression level of T cell functional markers (IFN-γ and GrZB) in CD3^+^ T cells and CD80^+^ CD86^+^ DCs were measured using flow cytometry. Following the instructions of the Biotech ELISA kit, the supernatants of the co-culture system were collected to quantify the levels of secretory cytokines IFN-γ and IL-2.

To evaluate the indirect DC-to-T immune activation induced by Bio-HCP@FM-NPs, mature BMDCs were plated at 5 × 10⁵ cells/well in 6-well plates and maintained under culture conditions with PBS, Bio-HCP-NPs, Bio-HCP@EM-NPs, Bio-HCP@STCM-NPs or Bio-HCP@FM-NPs (150 μg/mL, each) for 48 h. Flow cytometry was employed to evaluate DC maturation. Thereafter, the treated BMDCs were co-cultured 1 × 10^6^ spleen cells for 72 h, and then cells and supernatant were collected. The cells were resuspended in FACS buffer and T cell activation was evaluated by flow cytometry and IFN-γ ELISA.

### Evaluation of fusion membranes

For confocal imaging, DiO (MCE, HY-D0969) and DiI (MCE, HY-D0083) dyes were used to label EM-NPs and STCM-NPs, and then fluorescent nanoparticles were co-extruded ≥ 30 times through a 100 nm membrane. The images were observed on a confocal laser scanning microscopy (Nikon EZ-C1 Si, Japan).

### SDS-PAGE of FM-NPs

10 µg of EM-NPs, STCM-NPs, and FM-NPs were mixed with a loading buffer (Invitrogen, Carlsbad, CA, USA) to reach a final volume of 20 µL. The specimens were subsequently heated to 100 °C for 10 min. Subsequently, they were loaded onto NuPAGE Novex 12% separating gels (Invitrogen) in MOPS running buffer (Invitrogen). Following 90 min of electrophoresis (30 min at 80 V and 60 min at 120 V), the gel was stained using Coomassie brilliant blue G250 (Beyotime, ST031). Then, the gel was photographed by a Multifunctional imaging system (Servicebio, SCG-W5000).

### RNA sequencing

After treatment with various membrane nanoparticles for 48 h, BMDCs were collected and sent to Beijing Novogene Technology Co., Ltd. for RNA sequencing.

### Western blot assay

The protein sample was extracted from DCs treated with PBS, Bio-HCP-NPs, Bio-HCP@EM-NPs, Bio-HCP@STCM-NPs, or Bio-HCP@FM-NPs for 72 h. Protein extracts were fractionated via SDS-PAGE (Thermo Fisher Scientific, USA), transferred onto polyvinylidene difluoride membranes (Bio-Rad, Hercules, CA, USA), and incubated with anti-NF-κB p65 (1:500, Abcam, ab207297), anti-NF-κB p-p65 (phosphor-S536) (1:300, Abcam, ab239882), anti-GAPDH (1:500, Abcam, ab8245). Reversible ponceau staining was utilized (Servicebio, Wuhan, China) to ensure equal protein loading and normalize the gels.

### Cytotoxicity measurement

To enable cell attachment, B16F10 and LCC cells (2 × 10^5^/well) were planted onto a 24-well micro-plate overnight. Subsequently, the initial culture media was substituted with fresh DMEM or RPMI 1640 solutions containing Bio-HCP-NPs and Bio-HCP@FM-NPs (150 μg/mL, each). The next day, to visually observe the killing effects, cells were stained with the live/dead staining kit (Beyotime, C2030S) for 30 min, washed with PBS three times, and then imaged using a laser confocal microscope (Nikon EZ-C1 Si, Japan).

### Mice and tumor models

Six-week-old female *C57BL/6* mice were acquired from Wuhan Shubeili Biology Science and Technology Co., Ltd. The researchers were granted IACUC Number 3672 for the year 2023 by the Institutional Animal Care and Use Committee at Tongji Medical College, Huazhong University of Science and Technology.

To assess the anti-tumor effects of the Bio-HCP@FM-NPs vaccine, B16F10 cells (5 × 10^5^ cells/mL) were subcutaneously implanted in the right flank of *C57BL/6* mice. Approximately six days afterward, the size of the tumor increased to nearly 50-100 mm^3^. Subsequently, mice were randomly assigned to five distinct treatment groups (n = 12) and subcutaneously injected with following treatments: (1) PBS solution, (2) Bio-HCP-NPs, (3) Bio-HCP@EM-NPs, (4) Bio-HCP@STCM-NPs, and (5) Bio-HCP@FM-NPs (1.5 mg/mL, 100 μL per mouse, each formulation administered four times at 4-day intervals) (Figure 3B). Tumor progression (tumor volume) and tumor weight were recorded every two days for 19 days. Tumor volume was measured using the following equation: V = (width)^2^ × length/2. The tumor tissue was collected for flow cytometry at 23 days after tumor implantation (n = 6). Subsequently, the mice were followed for survival monitoring and euthanized when tumor volume increased to nearly 1500 mm^3^ (n = 6).

To investigate the combined effect of the Bio-HCP@FM-NPs vaccine and immune checkpoint inhibitors (ICIs), B16F10 cells (5 × 10⁵ cells/mL) or LLC cells (1 × 10⁶ cells/mL) were subcutaneously implanted in the right flank of C57BL/6 mice, which were then randomly assigned to four treatment groups (n = 6): (1) PBS solution; (2) anti-PD-1 (BioXcell, clone RMP1-14, 100 μg per mouse); (3) Bio-HCP@FM-NPs (1.5 mg/mL, 100 μL per mouse); and (4) combination therapy (Bio-HCP@FM-NPs+ anti-PD-1) (Figures 5A and 5I). The mice were subcutaneously administered four times at intervals of 4 days. The tumor volume and weight of mice were recorded every two days for 19 days, then the mice were followed for survival monitoring and euthanized when tumor volume increased to nearly 1500 mm^3^.

In the post-surgical tumor recurrence model, tumors were excised when their volumes were approximately 50-100 mm^3^ on the sixth day after tumor implantation, leaving a residual tumor mass of ~1% volume. Subsequently, the mice were randomly assigned to four distinct treatment groups (n = 6): (1) PBS, (2) Bio-HCP@FM-NPs (1.5 mg/mL, 100 μL per mouse), (3) anti-PD-1 (BioXcell, clone RMP1-14, 100 μg per mouse), and (4) Bio-HCP@FM-NPs combined with anti-PD-1 (Figure 6A). The mice were subcutaneously injected with various formulations (four times at intervals of 4 days). The tumor volume and weight were recorded every two days for 26 days. The mice were followed for survival monitoring and euthanized when tumor volume increased to nearly 1500 mm^3^.

### The biodistribution and biosafety evaluation of Bio-HCP@FM-NPs

To investigate the biodistribution of nanoparticles, we labeled the Bio-HCP-NPs with FITC-conjugated dyes. Then, Bio-HCP-NPs, Bio-HCP@EM-NPs, Bio-HCP@STCM-NPs, and Bio-HCP@FM-NPs (100 μL per mouse, 1.5 mg/mL, each) were subcutaneously injected into the subcutaneous B16F10-bearing *C57BL/6* mice. Major organs, including the heart, spleen, liver, lung, kidney, tumor, and lymph nodes were collected at 24 h post-injection. Flow cytometry was conducted to quantify the relative mean fluorescence intensity of different organs compared to the Bio-HCP-NP control group. To validate the retention ability of Bio-HCP@FM-NPs *in vivo*, we also subcutaneously injected Bio-HCP-NPs, Bio-HCP@EM-NPs, Bio-HCP@STCM-NPs, and Bio-HCP@FM-NPs (100 μL per mouse, 1.5 mg/mL, each) in subcutaneous B16F10-bearing mice. We collected their tumor tissues for flow cytometry analysis on days 1, 5, 10, and 15 post-injection.

To assess the biosafety of Bio-HCP-NPs for their potential application *in vivo*, the healthy *C57BL/6* mice received subcutaneous of Bio-HCP-NPs, Bio-HCP@EM-NPs, Bio-HCP@STCM-NPs, and Bio-HCP@FM-NPs (100 μL per mouse, 1.5 mg/mL, each). After 14 days, mice were sacrificed for blood biochemistry, complete blood count (CBC), and histological analysis of major organs (heart, spleen, liver, lung, and kidney) via H&E staining. The serum, which was separated from blood samples by centrifuging at 3500 rpm for 10 min, was used for the blood biochemistry analysis for alanine aminotransferase (ALT), aspartate aminotransferase (AST), blood urea nitrogen (BUN) and creatine (CR). The whole blood was used for blood routine examination.

### Evaluation of the Bio-HCP@FM-NPs-mediated tumor rejection mechanism via depletion of various immune cells *in vivo*

To investigate the improved tumor rejection due to Bio-HCP@FM-NPs, we depleted key immune cells involved in anti-tumor responses in the post-surgical tumor recurrence model (Figure 6F). After constructing the post-surgical tumor recurrence model, the mice were randomly assigned to five groups (n = 6): (1) Bio-HCP@FM-NPs (1.5 mg/mL, 100 μL per mouse), (2) anti-Ly6G (1A8, 5 μg/injection every 2 days) + Bio-HCP@FM-NPs, (3) anti-CD19 (1D3, 5 μg/injection twice weekly)+ Bio-HCP@FM-NPs, (4) anti-CD4 (GK1.5, 200 μg/injection twice weekly)+ Bio-HCP@FM-NPs and (5) anti-CD8 (Lyt 2.1, 400 μg002Finjection twice weekly)+ Bio-HCP@FM-NPs. Antibodies were administered intraperitoneally to deplete cell subpopulations beginning a day before treatment with the Bio-HCP@FM-NPs-based vaccine. The following administration was done four times at 4-day intervals. The tumor volume and weight were recorded every two days for 26 days. Flow cytometry analysis of mononuclear cells from peripheral blood and spleens was conducted to measure the depletion efficiency of neutrophils, B cells, CD8^+^ T cells, and CD4^+^ T cells in mice. The mice were followed for survival monitoring and euthanized when tumor volume increased to nearly 1500 mm^3^.

### Flow Cytometry

To evaluate alterations in immune cells in the tumor microenvironment (TME), tumor samples were harvested and processed into single-cell suspensions. Red blood cells (RBCs) were lysed, followed by two washes with PBS and resuspension in PBS. Initially, samples were treated with the Zombie NIR™ Fixable Viability Kit (BioLegend, 423105) to eliminate non-viable cells. To identify cell surface markers, the samples were treated with anti-mouse antibodies for CD45 (BioLegend, 103137; 157214), CD3 (BioLegend, 100236), CD4 (BioLegend, 100509), CD8a (BioLegend, 100722), NK1.1 (BioLegend, 108732), CD11b (BioLegend, 101228), F4/80 (BioLegend, 123110), CD11c (BioLegend, 117317), Gr-1 (BioLegend, 108452), CD86 (BioLegend, 105011), and CD80 (BioLegend, 104707) at the suggested concentrations, followed by incubation at 4 °C for 30 min. To stain T cells for intracellular anti-IFN-γ (BioLegend, 505830) and anti-GrzB (BioLegend, 372208), cells were fixed and permeabilized after 4 h of stimulation at 37 °C with 5% CO_2_ using ionomycin calcium salt (100 ng/mL), monensin sodium salt (1.5 mg/mL), and phorbol 12-myristate 13-acetate (PMA; 100 ng/mL). For CD206 (BioLegend, 141706) staining, cells were also fixed and permeabilized.

### Statistical analysis

Data were processed using R statistical software (version 4.3.1). Data are shown as mean ± standard deviation (SD) of separate biological samples. The Student t-test was employed to compare the two groups. A one-way or two-way ANOVA followed by Bonferroni correction was used for multiple comparisons. Survival curves were evaluated using a log-rank (Mantel-Cox) test. The thresholds for statistical significance were established as: *p < 0.05, **p < 0.01, ***p < 0.001, ****p < 0.0001, with *ns* (not significant).

## Results and Discussion

### Synthesis and characterization of FM-NPs

**Figure 1:**
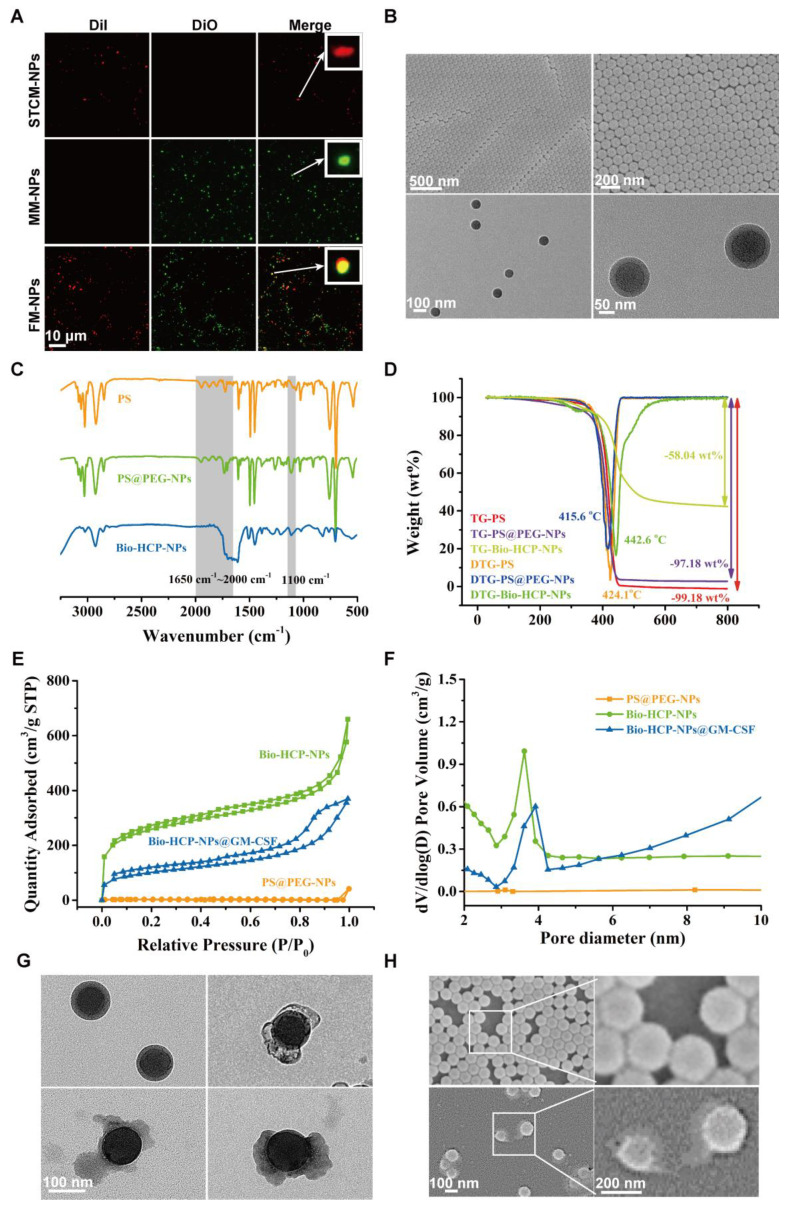
** Preparation and characterization of Bio-HCP@FM-NPs.** (A) Confocal images of STCM-NPs, EM-NPs and FM-NPs (The scale bar is 10 µm). (B) (Field Emission Scanning Electron Microscope) FESEM images and TEM (Transmission Electron Microscope) images of PS@PEG-NPs (The scale bar is 50 µm, 100 µm, 200 µm, 500 µm). (C) FT-IR spectral curves and surface chemical group analysis of polymer nanoparticles. (D) TGA and DTG curves of Bio-HCPs-NPs. (E) Isothermal N_2_ adsorption and desorption curves of Bio-HCPs-NPs before and after loading drugs. (F) Pore size distribution curves of Bio-HCPs-NPs before and after loading drugs. (G) TEM images of Bio-HCP-NPs, Bio-HCP@EM-NPs, Bio-HCP@STCM-NPs, and Bio-HCP@FM-NPs. Scale bars, 100 nm. (H) FESEM images of Bio-HCP@EM-NPs, Bio-HCP@STCM-NPs, and Bio-HCP@FM-NPs. Scale bars, 100 nm and 200 nm.

To produce potent cancer vaccines, it is crucial to design antigen carriers that act as immune boosters and transport tumor antigens into the cytoplasm of antigen-presenting cells (APCs) 11, 40, 41. Previous studies have shown that senescent tumor cell membranes (STCMs) possess more tumor-specific antigenic motifs compared to normal tumor cell membranes (TCMs) 17. Considering that vaccines based on TCMs have been broadly applied in cancer therapy, we hypothesized that the cancer vaccine based on the STCMs may be more effective than the normal cell membrane-based vaccine. Firstly, to verify the effectiveness of STCMs, the B16-F10 melanoma cells were cultured with different concentrations of cisplatin for 5 days to induce senescence in B16-F10 melanoma cells. Senescence-associated beta-galactosidase (SA*β*G) staining indicated that 200 nM of cisplatin effectively induced cellular senescence (Figure S1A). Thereafter, we investigated the ability of STCMs to induce DC maturation and activate T cells by measuring the expression levels of CD86, CD80, IFN-γ, and GZMB (Figure S1B). *In vitro*, BMDCs from *C57BL/6* mice were treated with different formulations (PBS, LPS, EMs, TCMs, and STCMs, each at 150 μg/mL) for 48 h. Cells treated with STCMs showed marked maturation (*p* < 0.0001) compared to other groups, as evidenced by upregulation of CD11c, MHC-I, MHC-II, CD80, and CD86 (Figures S1C-D). These findings indicate that STCMs can effectively enhance DC maturation. Pretreated DCs were co-cultured with spleen cells from mice for 72 h to explore the ability of STCMs to induce T cell activation *in vitro*. Compared to other groups, the proportion of effector CD8^+^ T cells markedly increased (*p* < 0.0001) in STCM-treated groups (Figures S1E-G). All these findings demonstrated that compared to TCMs, STCMs can more effectively activate the classic DC-to-T immunostimulatory route. Concerning the impressive immune-stimulating properties of STCMs and nanoscale tumor vaccines, which are readily absorbed by DCs, we processed the combined membranes of STCMs and EMs into nanoparticles to generate dual-functional hybrid membrane nanoparticles (FM-NPs). The SDS-PAGE revealed that FM-NPs contained the main proteins from EM-NPs and STCM-NPs (Figure S1H). STCM-NPs stained with DiI and EM-NPs dyed with DiO were shown as red and green nanoparticles in Figure 1A, while the fused FM-NPs were yellow ones, indicating a successful fusion of the two membrane-based nanoparticles. Fusion membranes obtained by extrusion were shown not to damage the individual membrane protein components. The mean sizes of EM-NPs, STCM-NPs, and FM-NPs were 97.97 ± 9.07 nm, 109.60 ± 6.09 nm, and 127.83 ± 9.91 nm, respectively (Figure S1I).

### Fabrication and characterization of Bio-HCP-NPs

Microporous or mesoporous materials are widely used as carriers in biomedical engineering and biotechnology owing to their high specific surface area, uniform pore characteristics, designable chemical units, and rich material categories. Despite advancements in drug delivery using conventional materials, like metal-organic frameworks (MOFs), covalent organic frameworks (COFs), and mesoporous silica nanoparticles (MSN), many challenges still limit their broader application, such as unstable chemical structures, high production costs, difficult surface modifications, and significant biotoxicity 34-36. Compared to these materials, hypercross-linked polymer nanoparticles (HCP-NPs) have uniform morphology, controllable size, low cost, and designable copolymers. The classical HCP-NPs are easily synthesized through two steps: 1) monodisperse polymer nano-colloids are prepared via emulsion polymerization 42; 2) microporous structures are formed via an external-knitting strategy 43, which has certain application potential in biomedical engineering and biotechnology. However, most HCP-NPs do not have a reasonable design to reduce their biotoxicity and are rarely applied as carriers of drugs. Therefore, we synthesized an internal/surface multi-functionalized hypercross-linked polymer material with classical core-shell structure, surface biocompatibility, and internal microporous properties.

Firstly, the core-shell-structural polystyrene@polyethylene glycol nanoparticles (PS@PEG-NPs) were synthesized via hydrophilicity differences between monomer molecules after emulsion polymerization (Graphical Abstract). TEM and FESEM were used to observe the core-shell structure and the uniform morphology of PS@PEG-NPs (Figure 1B). The size distribution of PS@PEG-NPs was also assessed based on dynamic light scattering (DLS). The average size was 91 nm, which was in line with the results of FESEM (Figure S2A). Due to precross-linking via DVB, the as-prepared PS@PEG-NPs became microporous through Friedel-Crafts alkylation reaction to produce the target Bio-HCP-NPs. Figure 1C showed the FT-IR spectral curves, demonstrating the success of the above-mentioned chemical reaction. Pure PS and PS@PEG-NPs exhibited four continuous benzene ring absorption peaks from 1650 cm^-1^ to 2000 cm^-1^, due to the vibrations of the benzene ring. Due to the chemical cross-linking between benzene rings, the characteristic peaks from 1650 cm^-1^ to 2000 cm^-1^ of Bio-HCP-NPs disappeared after hypercross-linking, suggesting the success of the internal Friedel-Crafts alkylation reaction. In addition, some characteristic peaks (such as 1100 cm^-1^ of -C-O-C-) appeared in PS@PEG-NPs and Bio-HCP-NPs, but not in pure PS, confirming the successful grafting of the functional groups. Thermogravimetric analysis (TGA) was conducted to characterize the degree of cross-linking of polymer colloids via the carbon skeleton content. The weight reduction for both uncross-linked pure PS and PS@PEG-NPs was almost 100 wt%. In contrast, Bio-HCP-NPs exhibited a weight loss of only 58 wt%, confirming the success of the hypercross-linking reaction (Figure 1D). The changes in surface functional groups also were reflected by hydrophilicity (Figure S2B). Compared to pure PS, the hydrophilicity of PS@PEG-NPs significantly increased because of the introduction of EG groups, evidenced by a decreased water contact angle (WCA). Conversely, WCA increased after the hydrophobic hypercross-linking reaction, but the nanoparticles maintained hydrophilicity and dispersibility in water. XPS measured the surface elemental content of materials within a depth of approximately 10 nm. The grafting of functional groups can be determined based on the O element content (Figure S2C and Table S1). The surface O element content significantly increased after introducing the PEG unit (up to 20.98 atom%) but decreased after hypercross-linking, suggesting the success of the above chemical reaction.

We measured the pore properties of microporous polymer nanoparticles using BET method (Brunauer-Emmett-Teller analysis; Figures 1E-F). The isothermal N_2_ adsorption-desorption curve of Bio-HCP-NPs conformed to a typical type IV curve. At low pressure (P/P_0_ < 0.1), the nitrogen adsorption amount rapidly increased, suggesting the presence of numerous microporous structures. The hysteresis loop at moderate pressure levels (0.3 < P/P_0_ < 0.8) suggested that the co-existence of mesopores in Bio-HCP-NPs. Pore size distribution curves revealed that the pores were predominantly micropores and mesopores (Figure S2D). The single point-specific surface area at P/P_0_ = 0.1 was up to ~1000 m²/g. The microporous characteristics of Bio-HCP-NPs were reflected in the total pore analysis and HK (Horvath-Kawazoe) pore size distribution curves (Figures S2E-F). The total pore analysis indicated no significant differences in mesopore area, while the micropores of Bio-HCP-NPs were concentrated at 0.42 nm. Table S2 presents more data about Bio-HCP-NPs. By adjusting the St/EG ratio, we prepared several Bio-HCP-NPs with different sizes to select the most suitable Bio-HCP-NPs. With the increase in the proportion of the EG segment, the size of Bio-HCP-NPs and the corresponding specific surface area significantly decreased (Figure S2G). Among the above groups, Bio-HCP-NPs synthesized based on the St/EG ratio of 90:10 (wt/wt) exhibited the optimal size and highest specific surface area while ensuring material biocompatibility (Figures S2H-I, Table S3). Due to the drug-carrying properties of Bio-HCP-NPs, we proposed a biomimetic strategy in which FM-NPs and GM-CSF were together loaded into Bio-HCP-NPs to produce a personalized Bio-HCP@FM-NP vaccine and overcome the limitations of current senescent tumor cell-based vaccines. TEM and FESEM images showed that Bio-HCP@FM-NPs exhibited a uniform spherical nanostructure with an inner core and an outer shell (Figures 1G-H).

### Bio-HCP@FM-NPs promoted DC maturation and T cell activation by co-transporting tumor antigens and adjuvants and activating the NF-κB pathway

**Figure 2:**
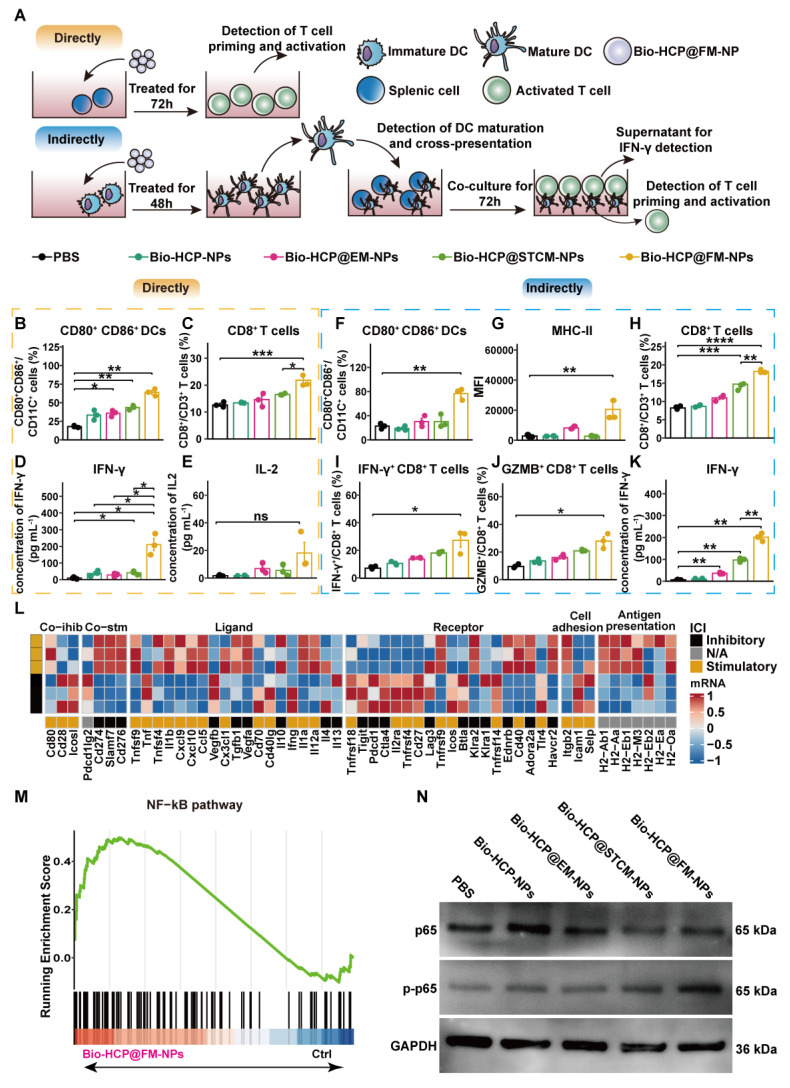
** Bio-HCP@FM-NPs deliver antigens and adjuvants to BMDCs and activate T cells through the NF-κB pathway.** (A) Diagram showing the dual T-cell activation assays in (B-K) (n = 3). The proportion of (B) mature DCs (CD11c^+^ CD80^+^ CD86^+^) and (C) CD8^+^ T cells after direct culture of spleen cells with PBS, Bio-HCP-NPs, Bio-HCP@EM-NPs, Bio-HCP@STCM-NPs, and Bio-HCP@FM-NPs for 72 h *in vitro*. (D) IFN-γ secretion and (E) IL-2 secretion in the supernatant of splenocytes directly treated with different nanoparticles for 72 h *in vitro*. (F-G) The percentage of DC maturation (CD11c^+^ CD80^+^ CD86^+^) and the levels of MHC-I and MHC-II on mDCs after indirect incubation with PBS, Bio-HCP-NPs, Bio-HCP@EM-NPs, Bio-HCP@STCM-NPs, and Bio-HCP@FM-NPs for 48 h *in vitro*. The proportions of (H) CD8^+^ T cells, (I) IFN-γ^+^ CD8^+^ T cells, and (J) GZMB^+^ CD8^+^ T cells indirectly co-cultured with DCs treated with nanoparticles (DC:T cell ratio 20:1). (K) IFN-γ levels in supernatants from indirect co-cultures (DC:T cell ratio 1:20) were measured by ELISA after 72 h. (L) Heatmap of RNA-seq data showing expression of 75 immunomodulatory genes in DCs treated with PBS or Bio-HCP@FM-NPs. (M) GSEA of the NF-κB pathway (n = 3). (N) Western blotting of the NF-κB pathway after 72 h of direct co-culture of PBS, Bio-HCP-NPs, Bio-HCP@EM-NPs, Bio-HCP@STCM-NPs, and Bio-HCP@FM-NPs with DCs. Data are presented as mean ± SD. One-way ANOVA with subsequent multiple comparison tests was conducted, where ns indicates no significance, *p < 0.05, **p < 0.01, ***p < 0.001, and ****p < 0.0001.

To evaluate the immune activation capability of Bio-HCP@FM-NPs, we conducted co-culture experiments to validate the direct activating effect of Bio-HCP@FM-NPs on DCs and T cells (Figure 2A). The exposure of splenocytes to Bio-HCP@FM-NPs (150 μg/mL) for 48 h notably increased the number of CD80^+^ CD86^+^ dendritic cells and CD8^+^ T cells, whereas the number of CD4^+^ T cells remained unchanged (Figures 2B-C and S3A-E). Comparable increases were detected in the concentrations of pro-inflammatory cytokines interferon-γ (IFN-γ) and IL-2 in the conditioned medium of splenocytes treated with Bio-HCP@FM-NPs (Figures 2D-2E). However, Bio-HCP@FM-NPs only modestly enhanced T-cell functions, possibly due to the lack of effective antigen presentation and high expression levels of agonist molecules. These results suggest that Bio-HCP@FM-NPs can act as nanoscale antigen-presenting cells (APCs), directly inducing cytotoxic CD8^+^ T cells.

To investigate whether Bio-HCP@FM-NPs can mediate indirect immune activation, we co-incubated BMDCs with Bio-HCP@FM-NPs (150 μg/mL) for 48 h (Figure 2A). The results showed a dramatic increase in DC activation markers (CD80, CD86, and MHC-II) in Bio-HCP@FM-NPs-stimulated BMDCs compared to Bio-HCP@STCM-NPs or Bio-HCP@EM-NPs (Figures 2F-G, S3A and 3F-I). Contrary to the immunogenic antigen moiety in Bio-HCP@STCM-NPs or Bio-HCP@EM-NPs, an enhanced immunostimulatory response was detected in response to Bio-HCP@FM-NPs, indicating the excellent ability of Bio-HCP@FM-NPs to promote DCs maturation. Thereafter, we co-cultured T cells with DCs pre-treated with Bio-HCP@FM-NPs at a 20:1 ratio for 72 h to confirm the efficiency of Bio-HCP@FM-NPs in activating naïve T cells. This treatment remarkably increased the number of CD3^+^ CD8^+^ T cells among splenocytes, and CD8^+^ T cells highly secreted IFN-γ and GZMB (Figures 2H-J). ELISA showed comparable patterns in IFN-γ release (Figure 2K). These results implied that Bio-HCP@FM-NPs can indirectly activate T cell function via promoting DC maturation. Subsequently, we collected the DCs pre-treated with Bio-HCP@FM-NPs for RNA-sequence. The RNA-seq results revealed increased levels of immune activation-related mRNAs and decreased levels of immunosuppression-related mRNAs in DCs treated with Bio-HCP@FM-NPs (Figure 2L). KEGG and GSEA analyses revealed the enrichment of NF-κB and cytokine-cytokine receptor interaction signaling pathways (Figures 2M and S4A). Considering the cellular NF-κB pathway of activation, we evaluated the effects of Bio-HCP@FM-NPs treatment on NF-κB signaling using Western blot analysis. As shown in Figure 2N, Bio-HCP@FM-NPs significantly upregulated the phosphorylation of NF-κB compared to other groups, indicating that Bio-HCP@FM-NPs can activate the NF-κB pathway. Previous studies have indicated that the IRF1 pathway, which relies on NF-κB, can enhance the maturation of cDC1, thereby promoting anti-tumor immune responses 44. These findings indicate that Bio-HCP@FM-NPs can enhance DC maturation and splenic T cell activation by co-transporting tumor antigens and adjuvants and activating the NF-κB pathway.

### Biodistribution and biosafety assessment of Bio-HCP@FM-NPs

The therapeutic efficacy of Bio-HCP@FM-NPs was significantly affected by lymph node targeting. Thus, we first evaluated the ability of Bio-HCP@FM-NPs to target dendritic cells. The Bio-HCP@FM-NPs were labeled with FITC fluorescent dye, and then dendritic cells were co-cultured with Bio-HCP@FM-NPs (150 μg/mL) for 24 h. The quantitative analysis of fluorescence signals showed that the group injected with Bio-HCP@FM-NPs exhibited the highest fluorescence intensity compared with the other groups, implying a remarkable capacity for targeting Bio-HCP@FM-NPs *in vitro* (Figure S5A). To further explore the targeting capability *in vivo*, the biodistribution of Bio-HCP@EM-NPs, Bio-HCP@STCM-NPs and Bio-HCP@FM-NPs was investigated by *in vivo* imaging 24 h after injection of Bio-HCP@EM-NPs, Bio-HCP@STCM-NPs or Bio-HCP@FM-NPs (1.5 mg/mL, 100 μL per mouse) in B16-F10 orthotopic tumor-bearing mice. The accumulation of fluorescence signals in the tumor and lymph nodes 24 h after Bio-HCP@FM-NPs treatment (Figures S5B-H) indicated that Bio-HCP@FM-NPs also had an excellent targeting ability *in vivo*. In addition, to evaluate the retention time of Bio-HCP@EM-NPs, Bio-HCP@STCM-NPs or Bio-HCP@FM-NPs, tumor tissues were harvested from orthotopic B16-F10 tumor-bearing mice at 1, 5, 10, and 15 days following Bio-HCP@EM-NPs, Bio-HCP@STCM-NPs or Bio-HCP@FM-NPs (1.5 mg/mL, 100 μL per mouse) injection. As shown in Figure S5I, the retention time of Bio-HCP@FM-NPs in tumors exceeded 15 days, confirming their long-term aggregation at the targeted location following a single dose of Bio-HCP@FM-NPs. Overall, these results indicated that Bio-HCP@FM-NPs had excellent targeting abilities both *in vitro* and *in vivo*.

The biosafety of Bio-HCP@FM-NPs was evaluated *in vitro* or *in vivo* to validate the feasibility of Bio-HCP@FM-NPs for treating cancer. The toxicity of Bio-HCP@FM-NPs was determined in mouse Lewis lung carcinoma (LLC) and melanoma (B16-F10) cells after a 24 h incubation using CCK-8 and apoptosis assays. The cell apoptosis assay showed that compared to the control group, Bio-HCP@FM-NPs only induced the apoptosis of a few cells in both cell lines (Figure S6A). Besides, the cell viability of B16-F10 cells and LLC cells was 92% and 90% after the cells were treated with Bio-HCP@FM-NPs (150 μg/mL) for 72 h, respectively (Figure S6B). These findings showed that Bio-HCP@FM-NPs exhibited no noticeable cytotoxic effects on cultured cells. To further assess the biosafety of nanovaccine *in vivo*, healthy mice (n = 3 per group) were given PBS (100 μL per mouse), Bio-HCP-NPs (1.5 mg/mL, 100 μL per mouse), Bio-HCP@EM-NPs, Bio-HCP@STCM-NPs, and Bio-HCP@FM-NPs (1.5 mg/mL, 100 μL per mouse) to assess the *in vivo* toxicity of Bio-HCP@FM-NPs. We assessed the compatibility of various nanovaccine with biological tissues by employing hematoxylin and eosin (H&E) staining and analyzing blood biochemical indicators of healthy tissues. Two weeks after injection, samples from key organs, such as the heart, liver, spleen, lungs, and kidneys, were collected and prepared for H&E staining (Figure S6C). Histological analysis unveiled that Bio-HCP-based vaccines did not affect these organs and did not trigger systemic inflammation. Two weeks after the injection, we conducted both complete blood count and blood chemical analyses. The numerical evaluation of the full blood count and serum biochemical tests revealed no notable differences between the control group (PBS) and the treated group (Figure S6D). Furthermore, multiple doses of nanoparticles neither impaired liver/kidney function nor affected hematological parameters. Meanwhile, the results confirmed that Bio-HCP@FM-NPs were not toxic to mice after multiple treatments.

### Bio-HCP@FM-NPs vaccination efficiently inhibited tumor progression in mice bearing B16-F10 melanoma

**Figure 3:**
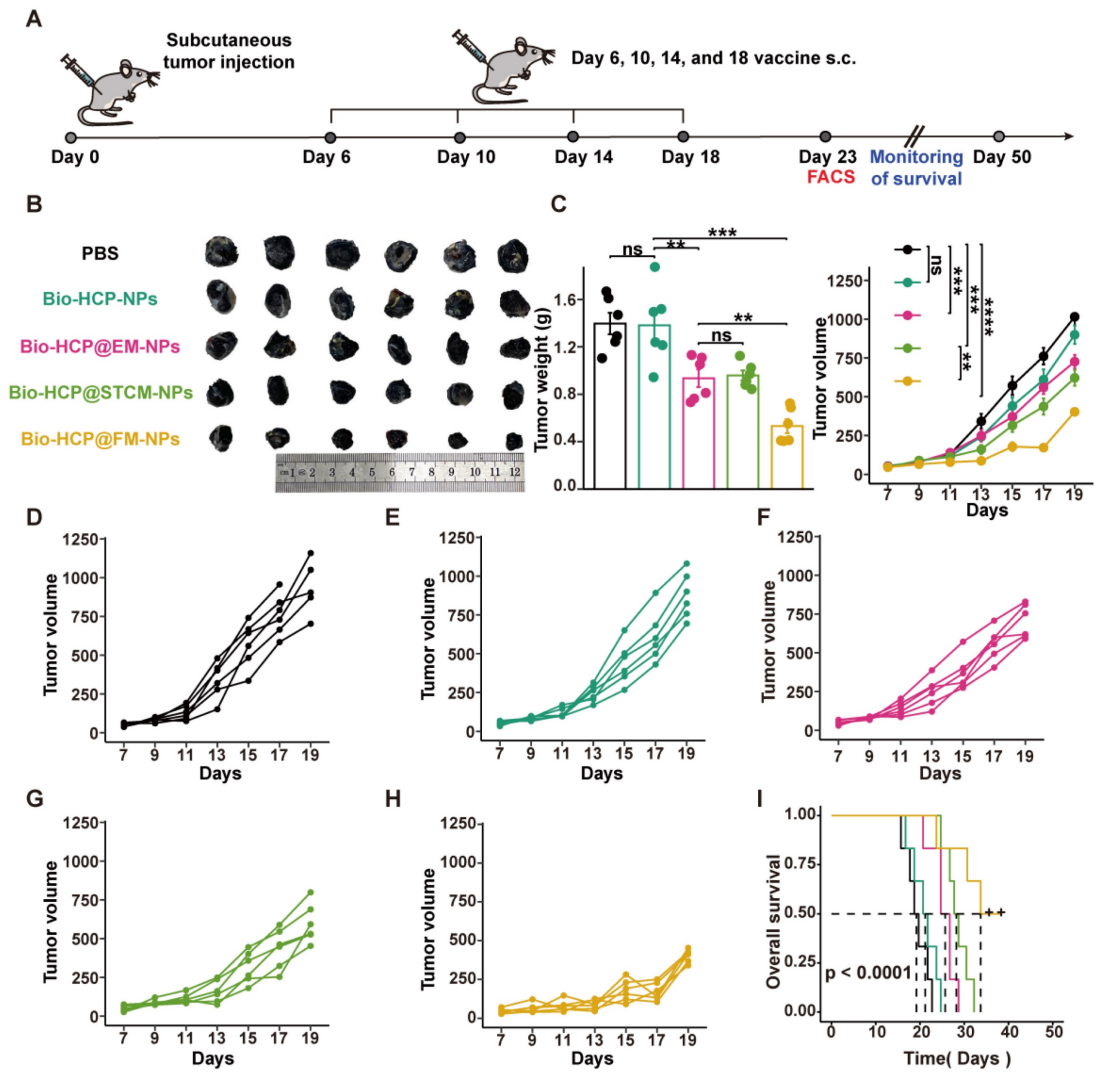
** Bio-HCP@FM-NP vaccination inhibited tumor growth in the murine B16-F10 tumor model.** (A) Schematic diagram showing the vaccine therapy of tumor-bearing mice. Mice received Bio-HCP@FM-NPs subcutaneously four times, 4 days apart (n = 6). (B) Sample images of excised tumors from B16-F10 tumor-bearing mice across various groups (PBS, Bio-HCP-NPs, Bio-HCP@EM-NPs, Bio-HCP@STCM-NPs, and Bio-HCP@FM-NPs, n = 6). (C) Tumor weight and growth curves for B16-F10 tumors in all groups (PBS, Bio-HCP-NPs, Bio-HCP@EM-NPs, Bio-HCP@STCM-NPs, and Bio-HCP@FM-NPs, n = 6). (D-H) Tumor growth trajectories for each mouse in different groups (PBS, Bio-HCP-NPs, Bio-HCP@EM-NPs, Bio-HCP@STCM-NPs, and Bio-HCP@FM-NPs, n = 6) of B16-F10 tumors. (I) Survival rates of mice across different groups over 50 days (n = 6). Data are presented as mean ± SD. One-way ANOVA with subsequent multiple comparison tests was conducted, where ns indicates no significance, **p < 0.01, ***p < 0.001 and ****p < 0.0001.

After verifying that Bio-HCP@FM-NPs can stimulate the differentiation of DCs and T cells *in vitro*, we assessed their effectiveness against tumors *in vivo* using the B16-F10 tumor model with low immunogenicity. After the subcutaneous injection of 1 × 10^6^ B16-F10 melanoma cells into mice for 6 days, we subcutaneously administered: PBS solution (100 µL); Bio-HCP-NPs (1.5 mg/mL, 100 μL per mouse); Bio-HCP@EM-NPs (1.5 mg/mL, 100 μL per mouse); Bio-HCP@STCM-NPs (1.5 mg/mL, 100 μL per mouse); or Bio-HCP@FM-NPs (1.5 mg/mL, 100 μL per mouse, containing 50 µg EM-NPs and 100 µg STCM-NPs) (Figure 3A). The Bio-HCP@FM-NPs group exhibited a marked reduction in tumor growth compared to the PBS and Bio-HCP groups (Figures 3B-C). Tumor growth was slightly controlled in the Bio-HCP@EM-NPs and Bio-HCP@STCM-NPs groups, whereas Bio-HCP@FM-NPs most effectively suppressed tumor growth. After 23 days of tumor inoculation, five out of six mice in the Bio-HCP@FM-NPs group had a tumor volume of less than 400 mm^3^ (Figures 3D-H). Survival analysis showed significant differences in survival rates, with the Bio-HCP@FM-NPs group exhibiting a 50% survival rate (3/6) (Figure 3I), better than that of the Bio-HCP@EM-NPs and Bio-HCP@STCM-NPs groups (*p* < 0.0001). Overall, these results suggested that Bio-HCP@FM-NPs inhibited tumor growth and improved survival.

### Anti-tumor immunity induced by Bio-HCP@FM-NPs *in vivo*

**Figure 4:**
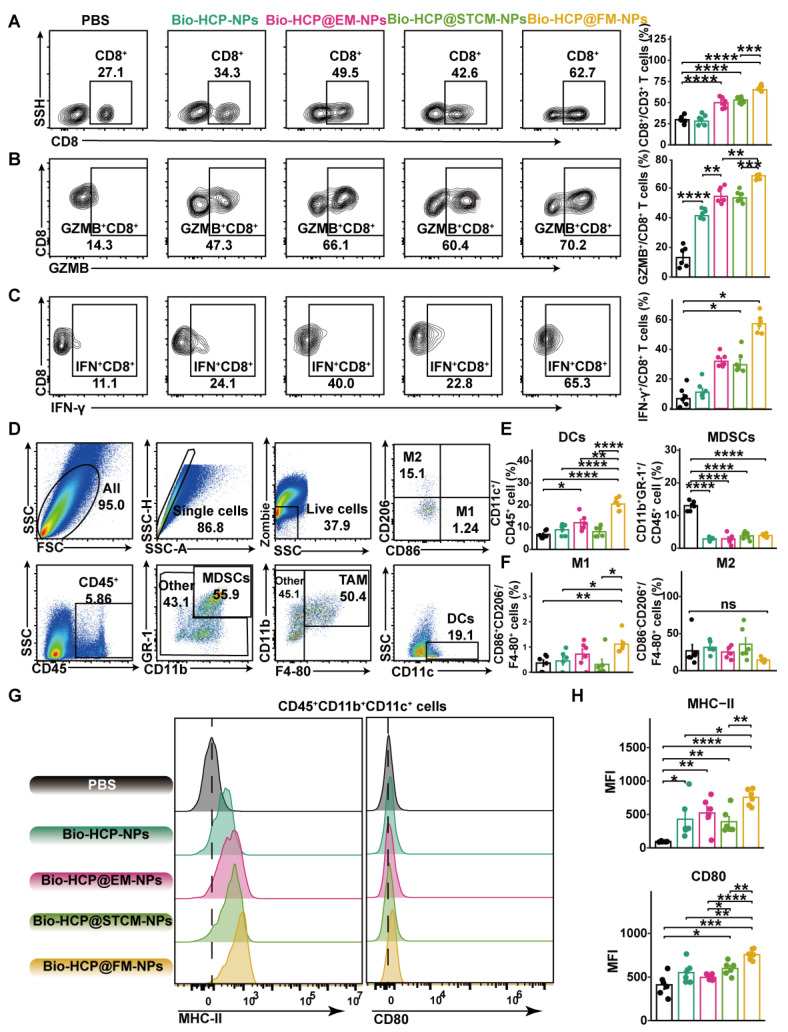
** Immune reactions against tumors triggered by Bio-HCP@FM-NPs vaccine.** (A) The percentage of CD8^+^ T cells was assessed using flow cytometry after co-culturing with various nanoparticles (PBS, Bio-HCP-NPs, Bio-HCP@EM-NPs, Bio-HCP@STCM-NPs, and Bio-HCP@FM-NPs, n = 6). (B-C) Sample flow cytometry plots of GZMB^+^ CD8^+^ T cells (top) and IFN-γ^+^ CD8^+^ T cells (bottom) in tumor samples (n = 6). (D) Strategy for flow cytometry gating to quantify the proportions of tumor-associated macrophages (TAMs), myeloid-derived suppressor cells (MDSCs), and dendritic cells (DCs) in tumor samples. (E) The percentages of DCs and MDSCs in tumor tissues (n = 6). (F) The proportion of M1 and M2 macrophages among TAMs (n = 6). (G) Representative flow cytometry images of MHC-II and CD80 on tumor-infiltrating DCs in different groups (n = 6). (H) The expression levels of MHC-II and CD80 on dendritic cells infiltrating tumors in various groups of *C57BL/6* mice (n = 6). Data are presented as mean ± SD. One-way ANOVA with subsequent multiple comparison tests was conducted, where ns indicates no significance, *p < 0.05, **p < 0.01, ***p < 0.001, and ****p < 0.0001.

To identify the anti-cancer immune reactions triggered by Bio-HCP@FM-NPs *in vivo*, mice were sacrificed five days after receiving four rounds of injection, and their tissues were collected for flow cytometry (Figure S7A). Compared to Bio-HCP@EM-NPs or Bio-HCP@STCM-NPs, Bio-HCP@FM-NPs notably increased the number of CD3^+^ T cells and CD8^+^ T cells in tumor tissues (Figures 4A and S7B); however, no significant changes were observed in the number of CD4^+^ T cells (Figure S7C). Interferon-γ (IFN-γ) and granzyme B (GZMB), two mediators of T cell-induced tumor-killing, were significantly upregulated in the Bio-HCP@FM-NPs group (Figures 4B-C and S7D-E). We also measured macrophage and DC activation in the TME (Figure 4D). Bio-HCP@FM-NPs significantly increased the abundance of DCs and reduced the abundance of MDSCs compared to other groups (Figure 4E). Although the proportion of M1 macrophages considerably increased after treatment with Bio-HCP@FM-NPs, the percentage of M2 macrophages remained largely unchanged (Figures 4F and S7F). APCs showed increased activation after exposure to Bio-HCP@FM-NPs, as evidenced by increased expression of CD80 and MHC-II on DCs (Figures 4G-H). These findings indicated that Bio-HCP@FM-NPs induced a robust proinflammatory response in DCs and enhanced the function of CD8^+^ cytotoxic T cells* in vivo*.

### Bio-HCP@FM-NPs enhanced the efficacy of immunotherapy with anti-PD-1 in multiple murine tumor models

**Figure 5:**
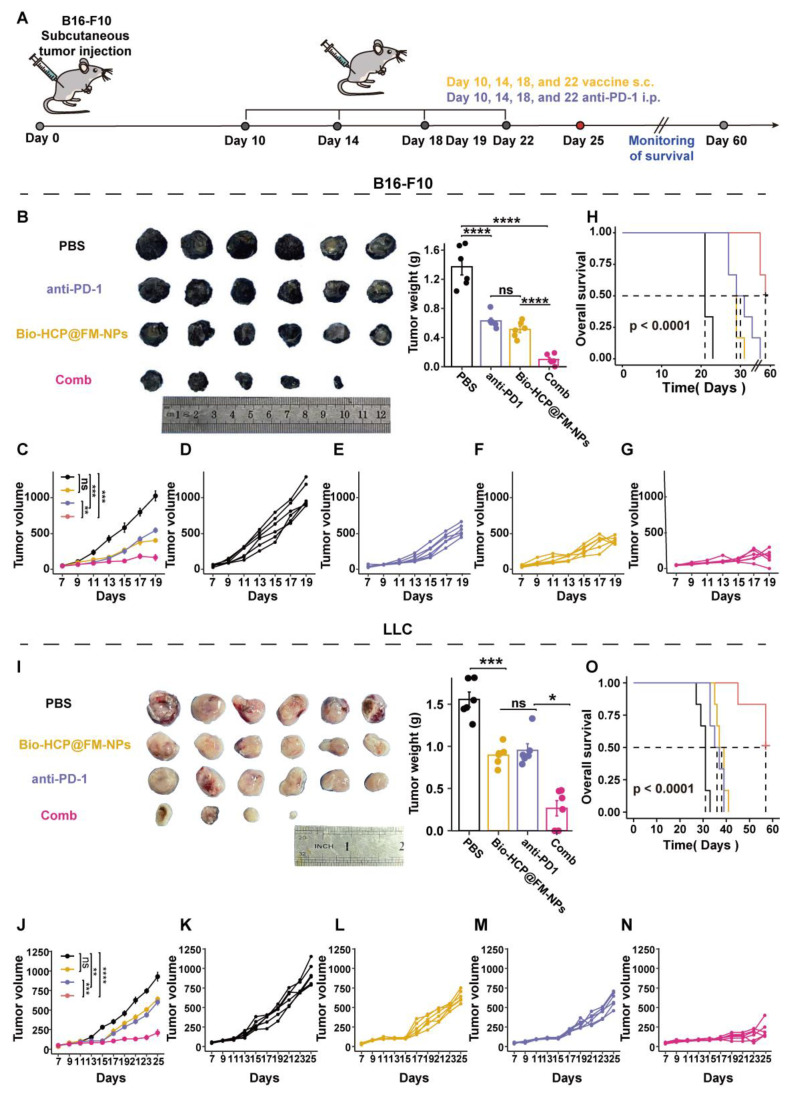
** Treatment with Bio-HCP@FM-NPs combined with anti-PD-1 exhibited robust anti-tumor effects.** (A) Postoperative treatment scheme in multiple tumor models (n = 6). (B) Sample images and the weight of excised tumors from B16-F10 tumor-bearing mice among groups (PBS, anti-PD-1 alone, Bio-HCP@FM-NPs, and Bio-HCP@FM-NPs + anti-PD-1 (Comb)). (C) Tumor progression curves for various groups of mice with B16-F10 tumors. (D-G) Individual growth curves of tumors for each mouse across various groups of B16-F10 tumor-bearing mice. (H) Kaplan-Meier plots depicting the survival rates of mice with B16-F10 tumors across various cohorts over 60 days. (I) Representative images and the weight of tumors harvested from LLC tumor-bearing mice in all groups. (J) Tumor growth curves of LLC tumor-bearing mice receiving different treatments (n = 6). (K-N) Growth trajectories of tumors for mice with LLC tumors in different groups (n = 6). (O) Survival curves of mice bearing LLC tumor in different groups over 60 days (n = 6). Data are presented as mean ± SD. One-way ANOVA with subsequent multiple comparison tests was conducted, where ns indicates no significance, *p < 0.05, **p < 0.01, ***p < 0.001, and ****p < 0.0001.

Although Bio-HCP@FM-NPs effectively inhibited tumor growth in the *C57BL/6* mouse model of B16-F10 tumors, tumors continued to grow slowly. Inspired by previous clinical and experimental results, we combined Bio-HCP@FM-NPs with anti-PD-1. Tumor volumes and the survival of the four groups of mice (PBS, Bio-HCP@FM-NPs (1.5 mg/mL, 100 μL per mouse), anti-PD-1 (BioXcell, RMP1-14, 100 μg per mouse), or Bio-HCP@FM-NPs + anti-PD-1 combination group) were monitored over 25 days (Figure 5A). Although anti-PD-1 alone had a limited effect on tumor suppression or survival, its combination with Bio-HCP@FM-NPs exhibited promising results (Figure 5B). On day 19, the average tumor volume in the Bio-HCP@FM-NPs + anti-PD-1 group was 212 mm^3^, compared to 455 mm^3^ in the Bio-HCP@FM-NP or 495 mm^3^ anti-PD-1 group (Figures 5C-G). The survival curve recapitulated these results. The Bio-HCP@FM-NPs + anti-PD-1 group exhibited a 66.7% survival rate (4/6) on day 37, but all mice in the PBS group died by day 23 (Figure 5H). To demonstrate the universality of Bio-HCP@FM-NPs in immunotherapy, we inoculated *C57BL/6* mice with LLC lung tumor cells and adopted a similar vaccination schedule (Figure 5A). The combination of Bio-HCP@FM-NPs and anti-PD-1 effectively inhibited tumor growth (Figure 5I). Using the LCC model, the mean tumor volume for both the Bio-HCP@FM-NP and anti-PD-1 groups was more than 661 mm^3^ by the 25 days after tumor implantation, whereas the Bio-HCP@FM-NPs + anti-PD-1 group showed a significantly lower average tumor volume of 240 mm³ (Figures 5J-N). The survival curve for mice with tumors displayed comparable outcomes. All mice in the Bio-HCP@FM-NP and anti-PD-1 groups died within 41 days after tumor inoculation, whereas the survival rate of mice in the Bio-HCP@FM-NP + anti-PD-1 group was 83.33% (5/6) at 60 days after tumor inoculation. In contrast, all mice in the PBS group died within 33 days (Figure 5O). Overall, our results suggest that Bio-HCP@FM-NP vaccines can enhance the efficacy of anti-PD-1 in several solid tumors.

### The integration of personalized Bio-HCP@FM-NPs with ICB successfully prevented the recurrence of tumors after surgery

**Figure 6:**
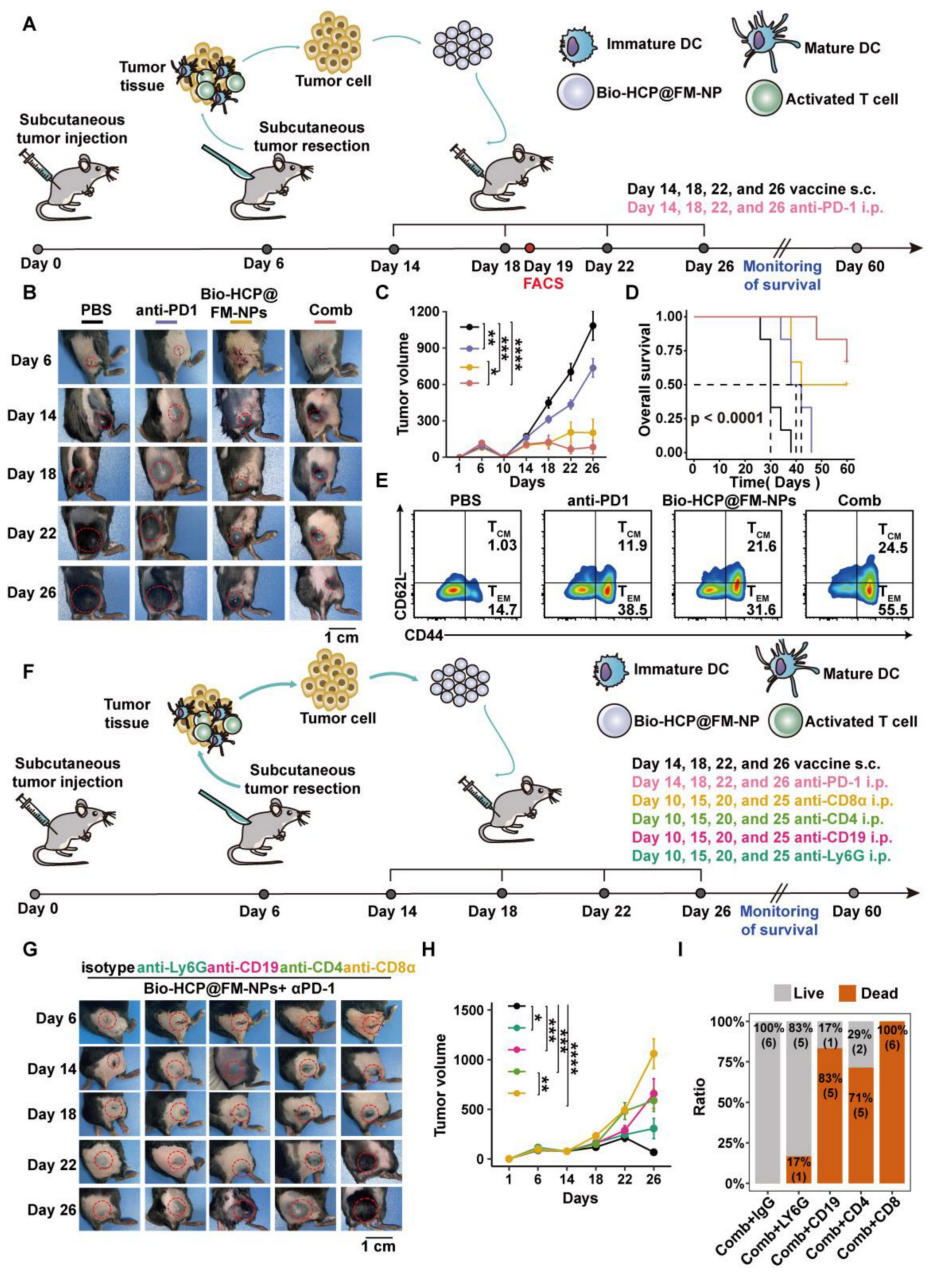
** The effectiveness of combined personalized Bio-HCP@FM-NP vaccination and immune checkpoint blockade therapy to prevent postoperative tumor recurrence.** (A) Postoperative treatment regimen for B16-F10 tumor-bearing mice (n = 6). (B-C) Sample tumor images and growth charts from mice with B16-F10 tumors subjected to various treatments (PBS, Bio-HCP@FM-NPs, anti-PD-1 alone, and Bio-HCP@FM-NPs combined with anti-PD-1 (Comb)). (D) Survival curves of tumor-bearing mice in different groups. (E) Sample flow cytometry images of TEM (CD44^+^ CD62L^-^) and TCM (CD44^+^ CD62L^+^) cells in tumor-draining lymph nodes. (F) Schematic illustration of the experimental design combining ICB therapy and Bio-HCP@FM-NP vaccine in postoperative tumor recurrence (n = 6). (G) Representative images of tumors and (H) tumor growth curves in different groups. (I) Survival rates of mice treated with depleting antibodies. Data are presented as mean ± SD. One-way ANOVA with subsequent multiple comparison tests was conducted, where ns indicates no significance, *p < 0.05, **p < 0.01, ***p < 0.001, and ****p < 0.0001.

Surgical intervention is the preferred treatment for patients with cancer, especially for those with advanced-stage cancer. Producing personalized cancer vaccines from a patient's tumor tissues has received much attention in the field of immunotherapy. Therefore, we evaluated the efficacy of postoperative treatment options (with close clinical relevance) using personalized Bio-HCP@FM-NPs across various tumor models. Visible melanomas were surgically removed 10 days after the inoculation of mice with B16-F10 or LLC cells (Figure 6A). The combination of subcutaneous Bio-HCP@FM-NPs and intraperitoneal anti-PD-1 almost completely prevented tumor recurrence and markedly decelerated the progression of remaining lesions (Figures 6B-C and S8A-C).

Improved responses to immune checkpoint blockade (ICB) therapy are shown in Figure 6D. Compared to those receiving only Bio-HCP@FM-NPs, significant survival benefit was observed for mice receiving Bio-HCP@FM-NPs plus anti-PD-1 (Bio-HCP@FM-NPs = 48 days; Bio-HCP@FM-NPs + anti-PD-1 = 58 days). Observing Bio-HCP@FM-NP-induced responsiveness to ICB, we investigated whether this tailored nanovaccine can provide strong and lasting memory T-cell immunity in conjunction with ICB. An increase in effector memory T cells (TEM: CD8^+^ CD44^+^ CD62L^-^) and central memory T cells (TCM: CD8^+^ CD44^+^ CD62L^+^) was observed in the peripheral blood of mice receiving tailored Bio-HCP@FM-NPs combined with ICB (Figure 6E). These findings indicated that the Bio-HCP@FM-NP vaccine triggered a potent anti-tumor response after tumor recurrence.

### Innate and adaptive immune responses are needed to ensure the efficacy of personalized Bio-HCP@FM-NP in preventing tumor recurrence

NK cells and macrophages play a crucial role in the innate immune response, whereas T and B cells are essential for the adaptive immune response 45. To investigate the improved tumor rejection mediated by Bio-HCP@FM-NPs, we eliminated key immune cells involved in anti-tumor responses, specifically CD4^+^ T cells, CD8^+^ T cells, B cells, and neutrophils (Figure 6F). These immune cells in the blood and spleen were inhibited after treatment with monoclonal antibodies (anti-CD8α, anti-CD4, anti-CD19, or anti-Ly6G). In contrast, treatment with immunoglobulin G (IgG) showed minimal effects (Figures S9A-B). The removal of CD4^+^ T cells, CD8^+^ T cells, or B cells greatly impaired the efficacy of Bio-HCP@FM-NPs combined with ICB in preventing tumor recurrence and improving survival. However, neutrophil depletion had a minor effect (Figures 6G-H and S10A-C). Mice receiving the Bio-HCP@FM-NP vaccine achieved 100% survival as long as their immune system was functional (Figure 6I). Nevertheless, all mice lacking CD8^+^ T cells experienced tumor regrowth even after being treated with Bio-HCP@FM-NPs. The decreased number of CD4^+^ T and B cells significantly shortened overall survival, whereas neutrophil depletion did not affect the efficacy of Bio-HCP@FM-NPs. Taken together, antibody depletion studies indicated that CD8^+^ T cells, CD4^+^ T cells, and B cells are critical for tumor rejection after vaccination. Other immune cell subsets, such as neutrophils, also contributed to tumor regression.

## Conclusion

We utilized senescent tumor cells to trigger anti-tumor immunity and improve cancer treatment. This study outlined the combination of bacterial inner membranes with surgically obtained STCMs to produce FM-NPs. These NPs delivered tumor antigens and activated DCs. Then, FM-NPs and GM-CSF were co-loaded into Bio-HCP-NPs to produce a personalized Bio-HCP@FM-NP vaccine. GM-CSF was rapidly released to attract naïve DCs to the nanovaccine. In addition, immature DCs (iDCs) were effectively activated by bifunctional FM-NPs, differentiating into mature DCs (mDCs) that were loaded with tumor antigens. Moreover, Bio-HCP@FM-NP vaccines, combined with anti-PD-1, demonstrated significant anti-tumor effects and prolonged survival in the mouse models of lung cancer and melanoma. Additionally, in lung cancer and melanoma resection and relapse models, the biomimetic individualized vaccine decreased tumor recurrence, prolonged the survival of tumor-bearing animals, and provided long-term tumor-specific protection. In summary, the Bio-HCP@FM-NP vaccine platform with intrinsic adjuvant properties can serve as a versatile platform to develop individualized cancer vaccines for diverse solid tumors, including breast and colorectal cancers.

## Supplementary Material

Supplementary figures and tables.

## Abbreviations

DCs: dendritic cells
LPS: lipopolysaccharides
PRRs: pattern recognition receptors
TM: tumor cell membrane
FMs: Fusion membranes
FM-NPs: FM nanoparticle vaccines
GM-CSF: granulocyte and macrophage colony growth factor
iDCs: immature DCs
mDCs: mature DCs
ATCC: American Tissue Culture Collection
FBS: fetal bovine serum
DLS: dynamic light scattering
BMDCs: bone marrow-derived dendritic cells
RT: room temperature
TME: tumor microenvironment
RBC: red blood cells
PMA: phorbol ester compound
ANOVA: single-factor
ANOVA SenTC: senescent tumor cells
IFN-γ: interferon-γ
GZMB: granzyme B
APC: antigen-presenting cell
ELISA: enzyme-linked immunosorbent assay
ICB: immune checkpoint blockade
NK: natural killer
IgG: immunoglobulin G
RBC: red blood cells
WBC: white blood cells
BUN: blood urea nitrogen
CR: creatinine
ALT: alanine transaminase
AST: aspartate aminotransferase
TDLN: tumor-draining lymph node
