# Supplementary material for: A bifunctional fusion membrane-based biocompatible nanovaccine to potentiate cancer immunotherapy
Source: Theranostics. 2025 Apr 21;15(12):5719–37. doi: 10.7150/thno.106376 (PMC12068300; doi:10.7150/thno.106376)
Supplement: Supplementary file 1 — Supplementary figures and tables. [file thnov15p5719s1.pdf]

A Bifunctional Fusion Membrane- Based Biocompatible Nanovaccine to Potentiate Cancer Immunotherapy

Wei Fu[a,f,1], *Xing Cai*[b,1], *Jinru Yang*[b,1], *Lian Yang*[c,d,*], *Yaoyu Pan*[e,*], *Zhan Tuo*[f,*]

[a]Chongqing Key Laboratory of Molecular Oncology and Epigenetics, The First Affiliated Hospital of Chongqing Medical University, Chongqing 400016, China.

[b]Department of Radiation and Medical Oncology, Zhongnan Hospital of Wuhan University, Wuhan 430071, P.R. China.

[c]Department of Radiology, Union Hospital, Tongji Medical College, Huazhong University of Science and Technology, Wuhan, 430022, China.

[d]Hubei Key Laboratory of Molecular Imaging, Wuhan, 430022, China.

[e]Department of Polymer, School of Material Science and Engineering, Hubei University, Wuhan, Hubei 430062, China.

[f]The Affiliated Cancer Hospital of Zhengzhou University & Henan Cancer Hospital, Zhengzhou 450008, China.

[*]E-mail address: tztuozhan@163.com (Z. Tuo).

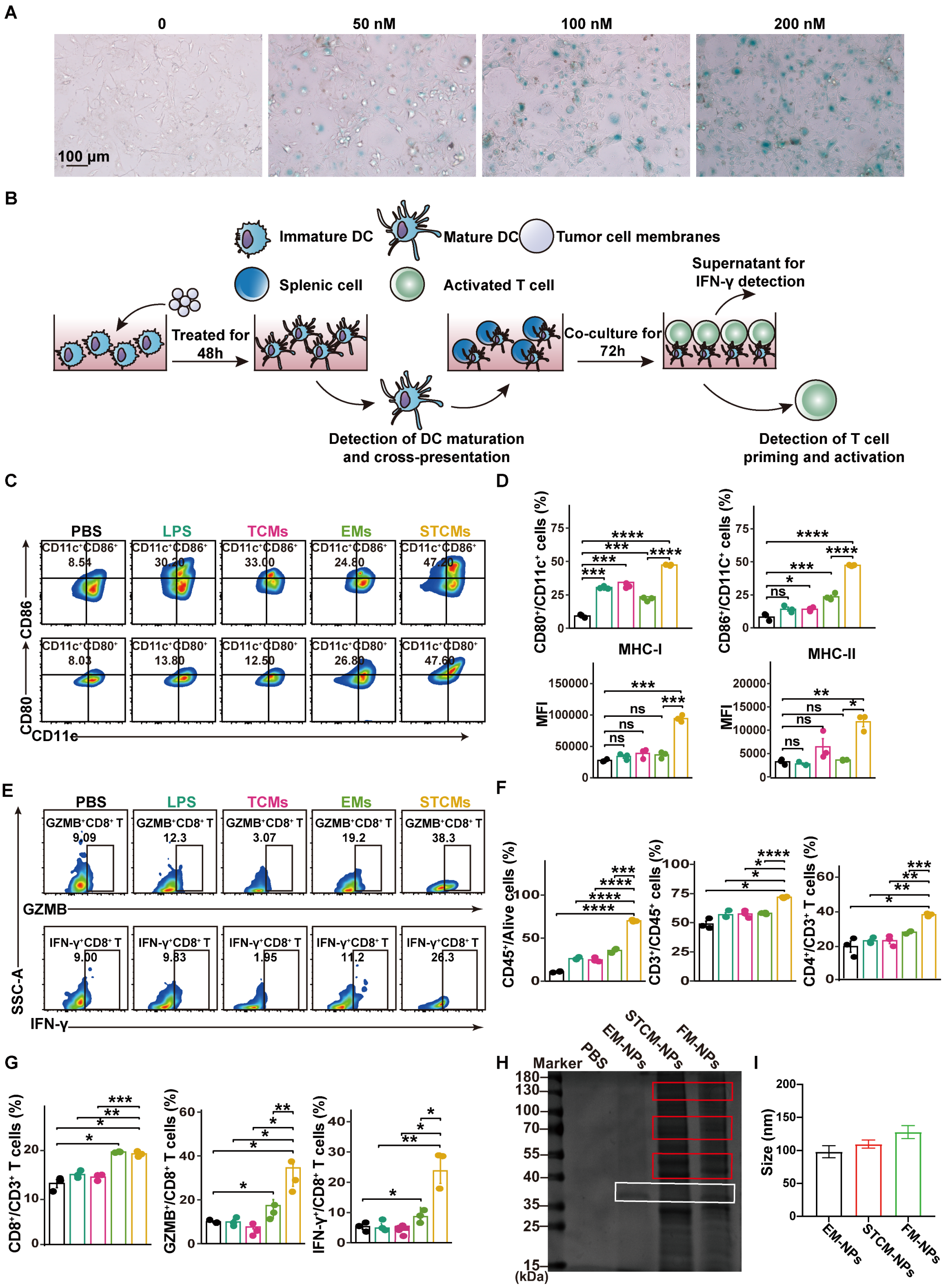

Supplementary Figure 1. STCM-NPs promote antigen presenting by DCs and activation of T cells, related to Figure 1. (A) SAβG staining of B16-F10 tumor cells following senescence induction via treatment with different concentrations of cisplatin for 6 days. Scale bar, 100 μm. (B) Sketch depicting the co-culture assays. (C)

Representative flow cytometry images of mDCs ($CD11c^+$ $CD80^+$ and $CD11c^+$ $CD86^+$) co-incubated with different groups (PBS, LPS, TCMs, EMs, and STCMs) for 48 h *in vitro* (n = 3). (D) The proportion of mDCs and the levels of MHC-I and MHC-II on mDCs after being co-cultured with various formulations for 48 h *in vitro* (n = 3). (E) Representative flow cytometry images of IFN-γ^+ $CD8^+$ T cells (lower) and $GZMB^+$ $CD8^+$ T cells (uper) after being co-cultured with BMDCs pre-treated with various formulations for 72 h *in vitro*. (F) Percentages of $CD45^+$ T cells, $CD3^+$ T cells and $CD4^+$ T cells (n = 3) after being co-cultured with BMDCs pre-treated various formulations for 72 h *in vitro*. (G) Percentages of $CD8^+$ T cells, $GZMB^+$ T cells and IFN-γ^+ $CD8^+$ T cells (gated from $CD3^+$ $CD8^+$ cells; n = 3). (H) The SDS-PAGE image of STCM-NPs (red rectangle), and EM-NPs (black rectangle). The amounts of protein in gels were 10 ug. (I) Diameter (n = 3) of EM-NPs, STCM-NPs and FM-NPs. Data are presented as mean ± SD. One-way ANOVA with subsequent multiple comparison tests were conducted, where ns indicates no significance, *$p < 0.05$, **$p < 0.01$, ***$p < 0.001$ and ****$p < 0.0001$.

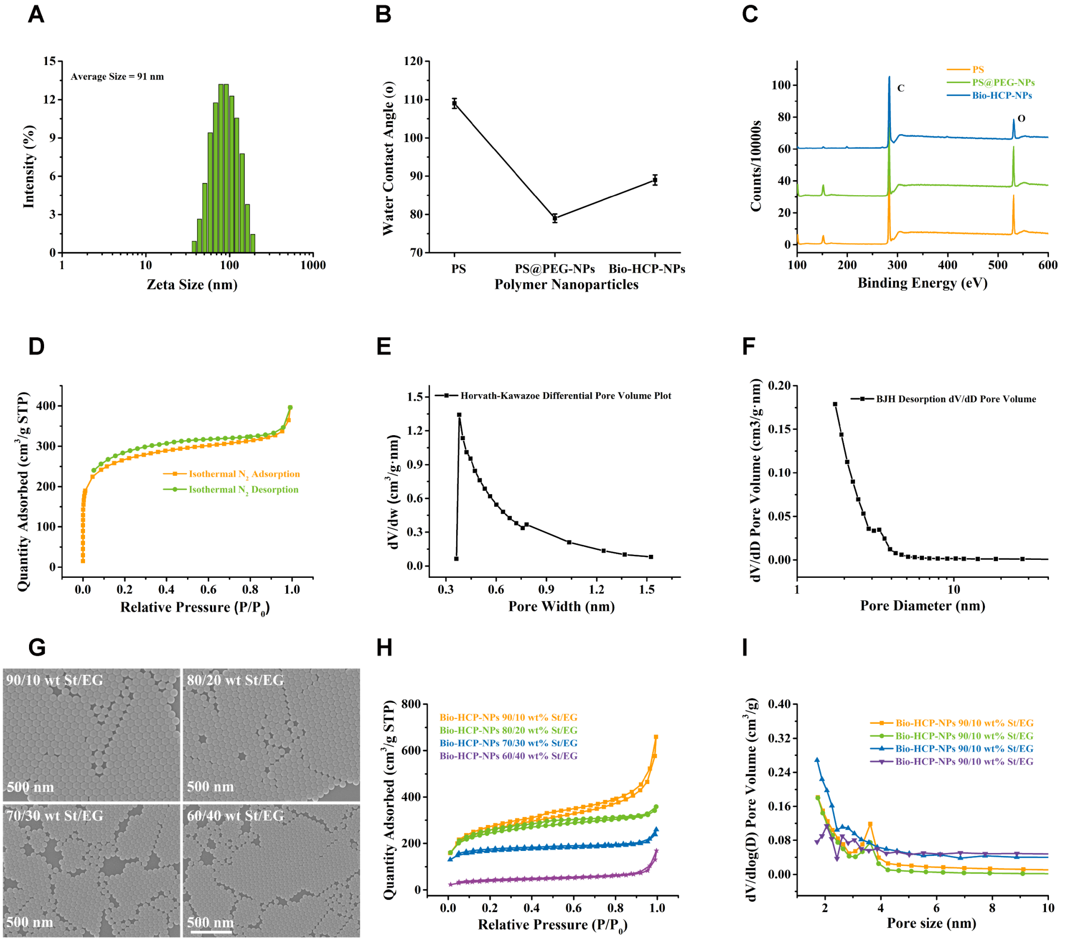

Supplementary Figure 2. Additional analysis and characterization of Bio-HCP-NPs. (A) Zeta size curves of Bio-HCP-NPs. (B) WCA analysis of PS, PS@PEG-NPs and Bio-HCP-NPs (C) XPS surface elements analysis of PS, PS@PEG-NPs and Bio-HCP-NPs. (D) Isothermal N2 absorption and desorption curves of Bio-HCP-NPs in total-pore BET testing. (E) Horvath-Kawazoe differential pore volume plot of Bio-HCP-NPs in total-pore BET testing. (F) BJH pore size curves of Bio-HCP-NPs in total-pore BET testing. (G) FESEM images of series Bio-HCP-NPs formed with different proportion of monomers. (H) Additional morphological and structural characterization of Bio-HCP-NPs in 90/10 wt% St/EG ratio. (I) Pore sizes distribution curves of series Bio-HCP-NPs formed with different proportion of monomers.

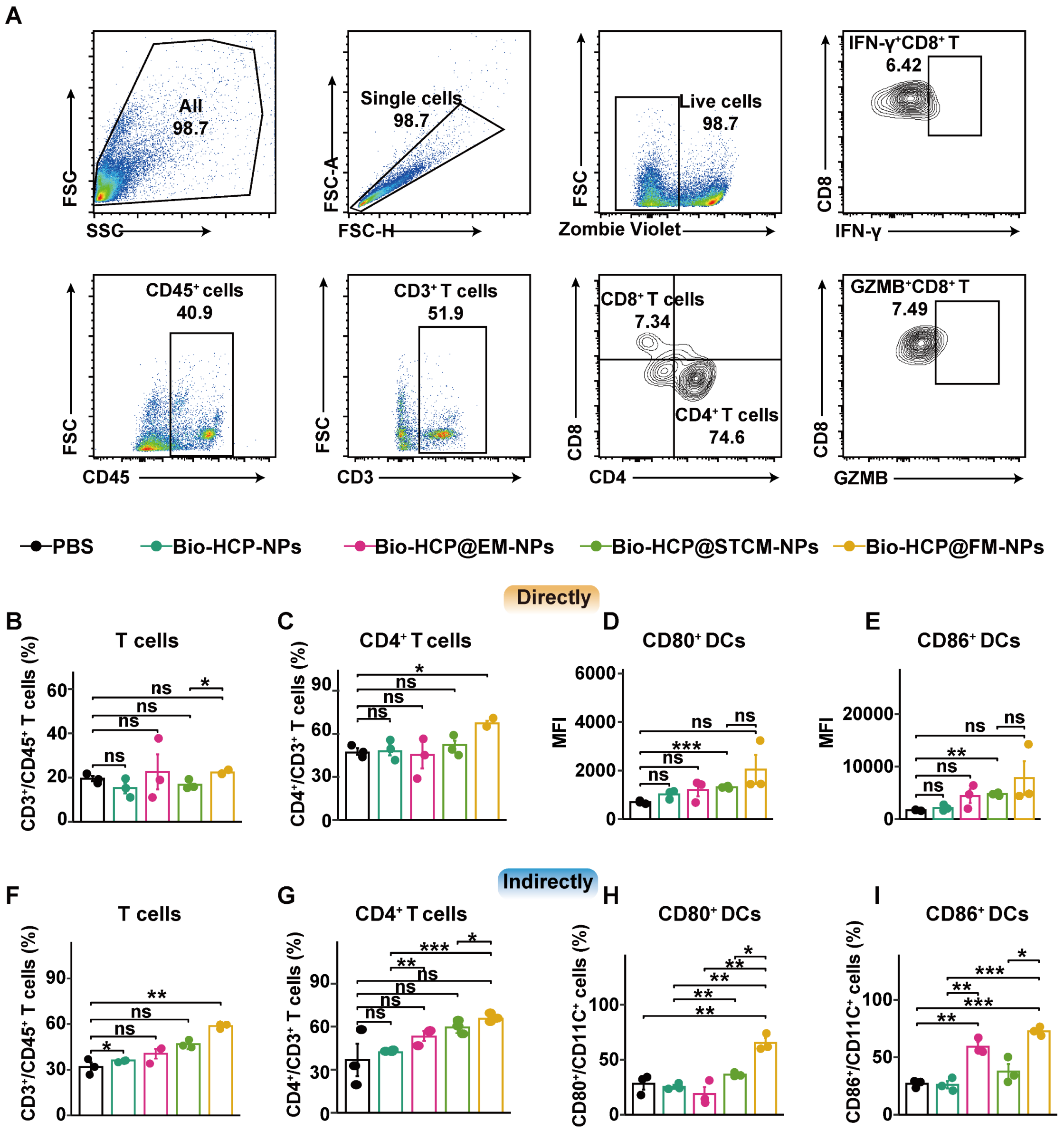

Supplementary Figure 3. Bio-HCP@FM-NPs activate DCs, related to Figure 3. (A) Flow cytometry gating strategy for measuring the percentages of T cells, IFN-γ^+ T cells and GZMB$^+$ T cells (n = 3). (B-C) Percentages of T cells, CD4$^+$ T cells in direct co-culture assay (n = 3). (D-E) Expression of CD86 and CD80 on DCs in direct co-culture assay (n = 3). (F-I) Percentages of T cells, CD4$^+$ T cells, CD80$^+$ DCs and CD86$^+$ DCs in indirect co-culture assay (n = 3).

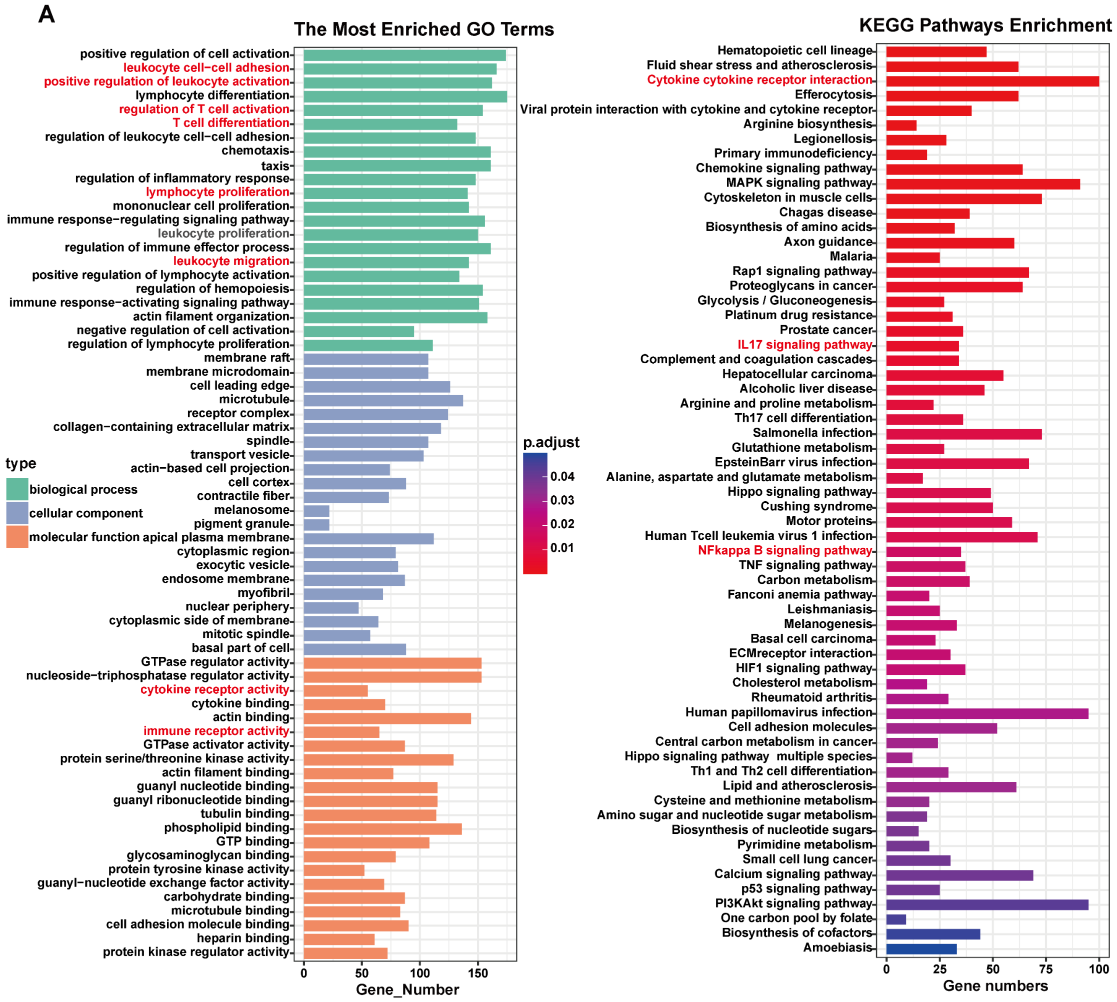

Supplementary Figure 4. GO and KEGG analysis of BMDCs, related to Figure 3.

(A) GO and KEGG analysis of BMDCs.

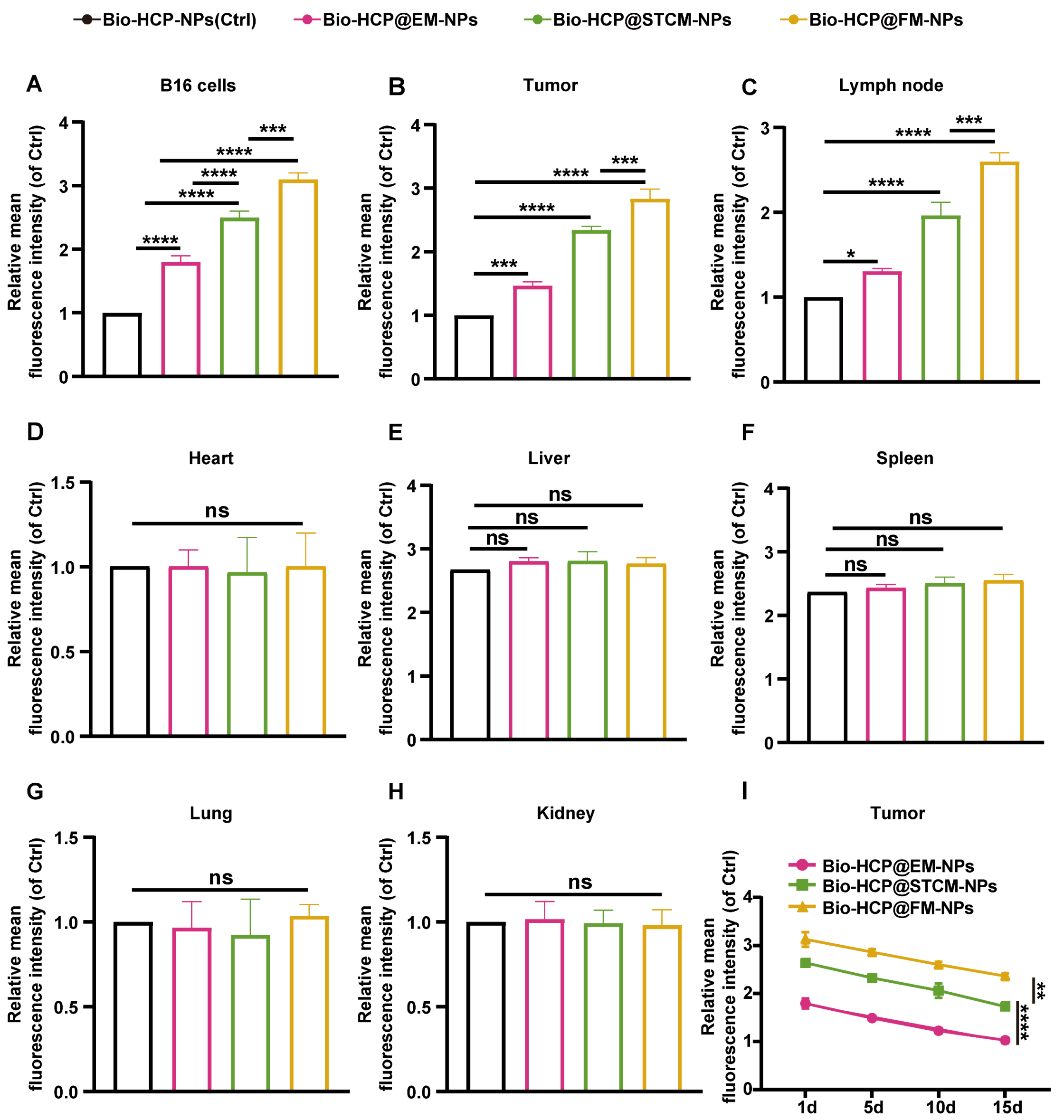

Supplementary Figure 5. Assessment of biodistribution and targeting capability of Bio-HCP@FM-NPs vaccination in *vitro* and *vivo*. (A) Quantitative analysis of fluorescence signals from FITC-labeled nanoparticles in DCs at 24 h post-injection. (B-H) The quantitative analysis of nanoparticle accumulation in major organs, tumor and lymph nodes from Bio-HCP@EM-NPs, Bio-HCP@STCM-NPs and Bio-HCP@FM-NPs injected B16-F10 cells orthotopic tumor-bearing mice *in vivo*. (I) Retention ability of Bio-HCP@EM-NPs, Bio-HCP@STCM-NPs and Bio-HCP@FM-NPs in tumor tissues of B16-F10 cells orthotopic tumor-bearing murine model quantified by flow

cytometry at different time points. Data are presented as mean ± SD, with n = 3 per group. Statistical significance is indicated as follows: n.s. (no significance), *$p < 0.05$, **$p < 0.01$, ***$p < 0.001$, ****$p < 0.0001$.

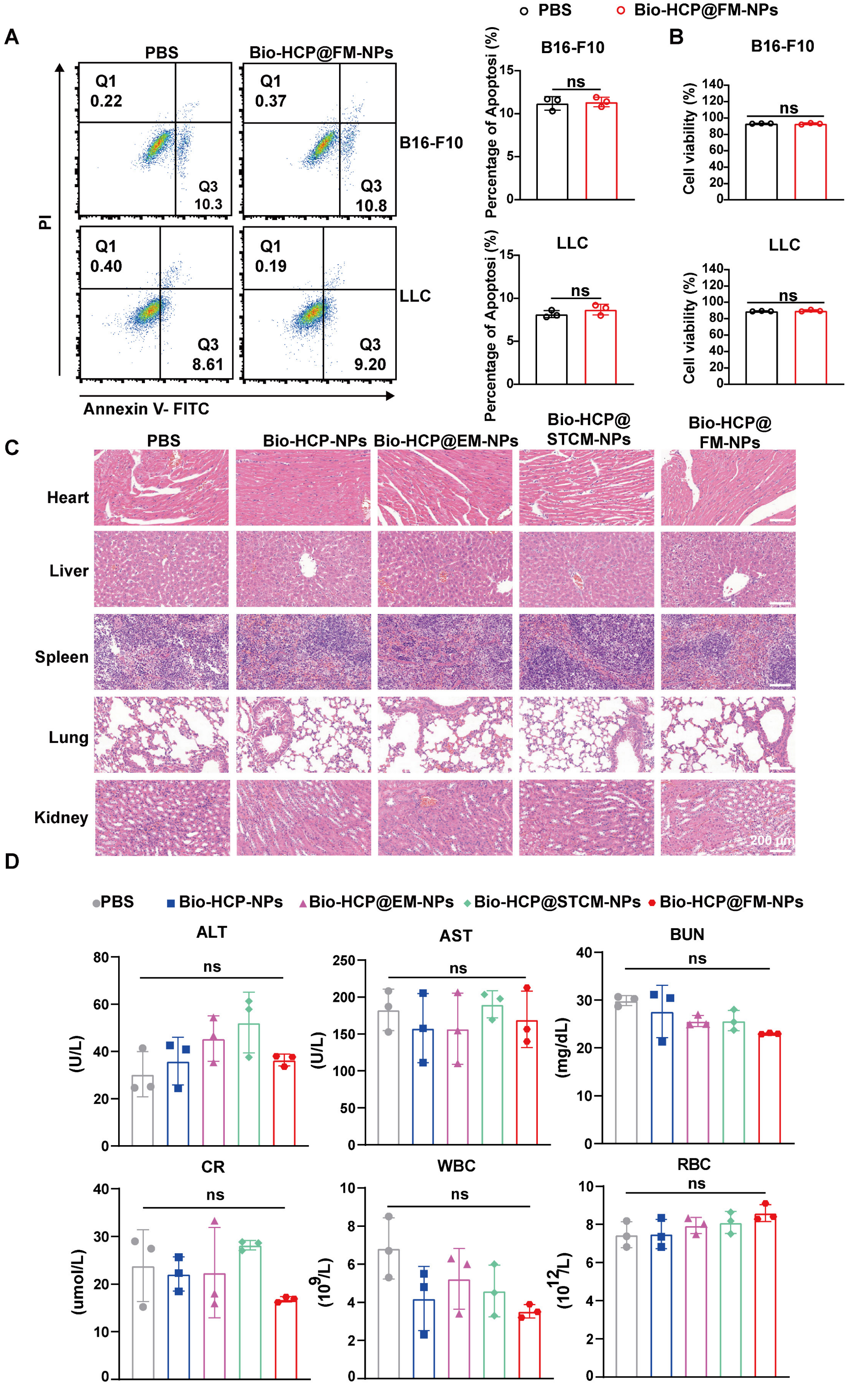

Supplementary Figure 6. Bio-HCP@FM-NPs show good biosafety. (A-B) The

cytotoxicity of Bio-HCP@FM-NPs against B16-F10 and LLC cells after co-incubation with nanovaccines. (C) H&E staining of heart, liver, spleen, lung, and kidney in B16-F10 mouse melanoma tumor model on day 7 after treatment. The scale bar is 200 μm. (D) Hemanalysis was performed on blood drawn from mice on day 7 after treatment. Aspartate transaminase (AST), white blood cell (WBC), alanine transaminase (ALT), red blood cell (RBC), blood urea nitrogen (BUN), and creatinine (CR) are presented as the means ± SEM. All data are mean ± SD; n = 3. *p <0.05, **p < 0.01, ***p < 0.001, and ns, not significant.

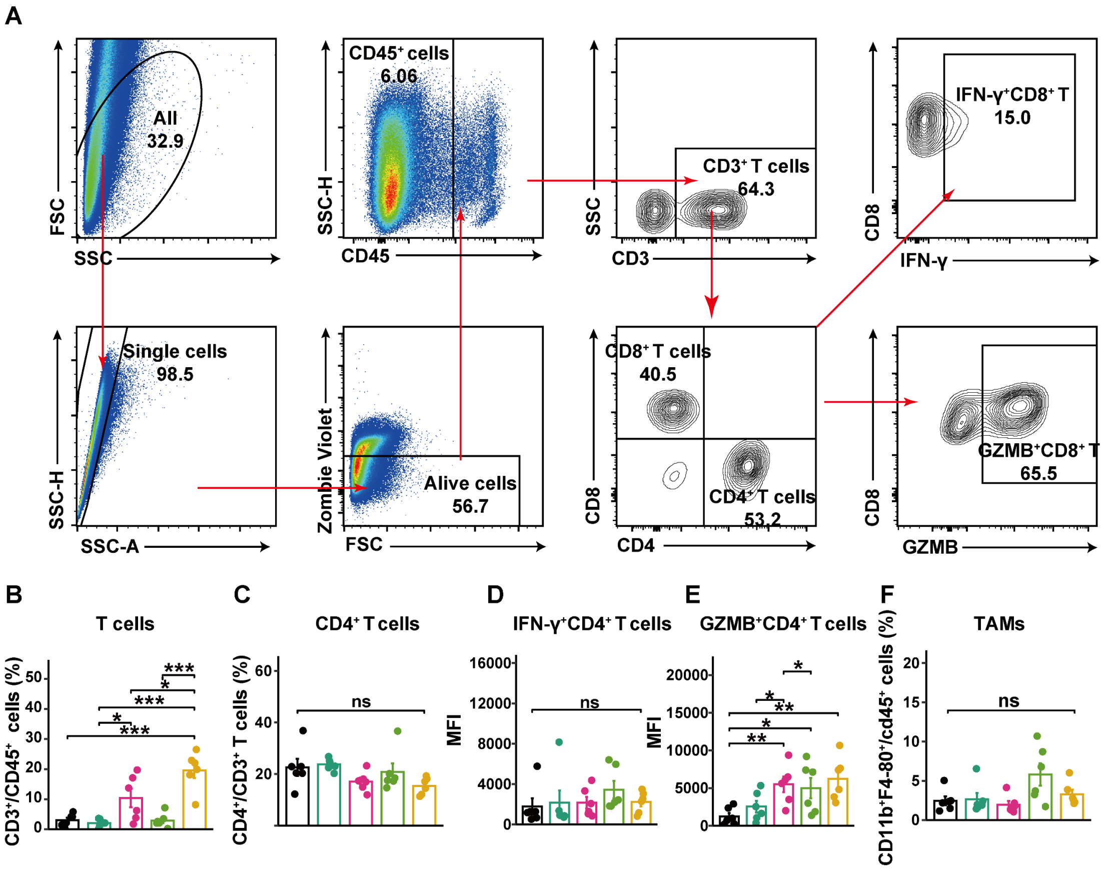

Supplementary Figure 7. Bio-HCP@FM-NPs increase tumor immune response in the TME, related to Figure 5. (A) Flow cytometry gating strategy for measuring the percentages of T cells, IFN-γ^+ and GZMB$^+$ T cells in tumor tissue. (B-C) Percentages

of T cells and CD4$^+$ T cells in tumor tissue. (D-E) Expression of IFN-γ and GZMB on CD4$^+$ T cells in tumor tissue (n = 3). (F) Percentages of TAMs in tumor tissue.

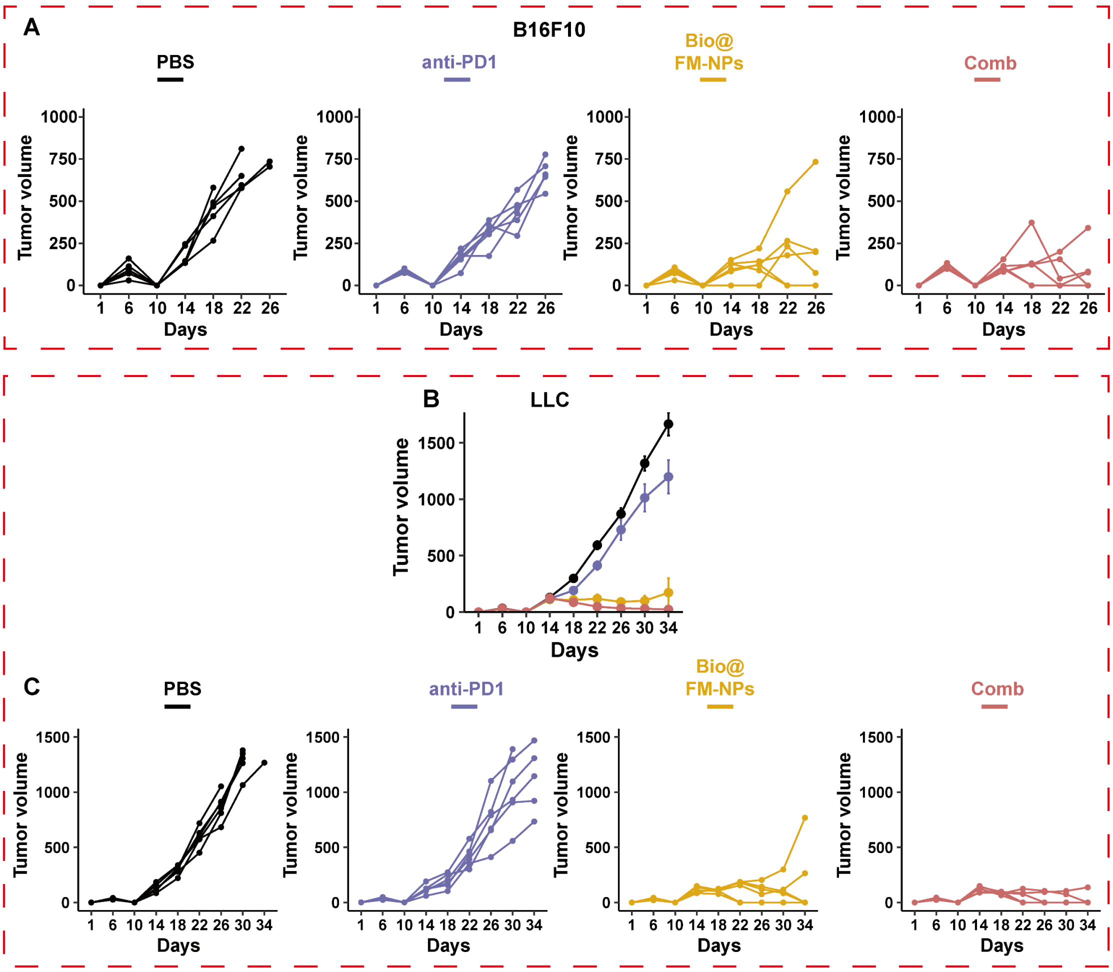

Supplementary Figure 8. Combinatorial efficacy of personalized Bio-HCP@FM-NPs vaccination and anti-PD-1 against postsurgical tumor recurrence, related to Figure 7.

(A) B16F10 tumor-growth curves for each mouse in different groups (n = 6). (B) Average tumor-growth curves of *C57BL/6J* mice bearing LLC tumor with different treatments as indicated (n = 6). (C) LLC tumor-growth curves for each mouse in different groups (n = 6).

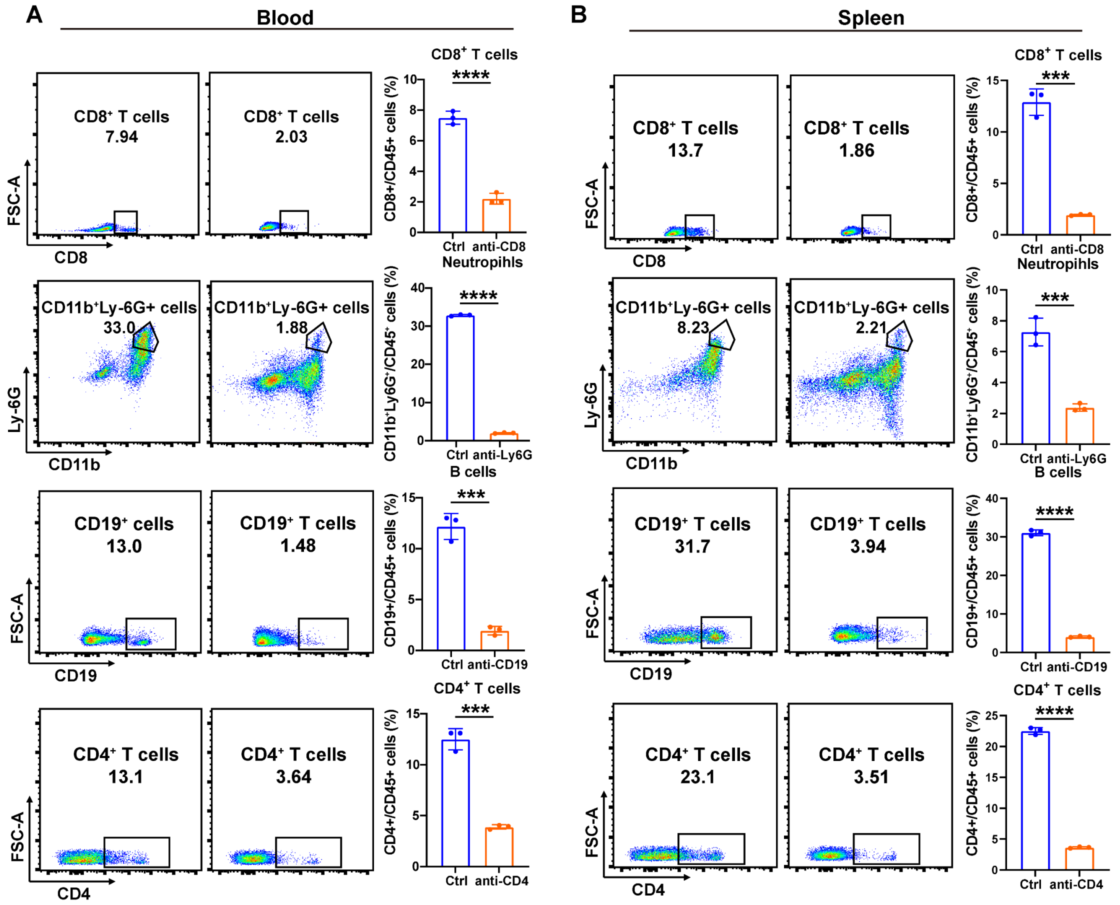

Supplementary Figure 9. Validation of the immune cell depletion efficiency, related to Figure 7. (A-B) Flow cytometry analysis of changes in immune cells from peripheral blood and spleen of mice subjected to different immune cell depletion treatments as indicated (n = 6-8 per group).

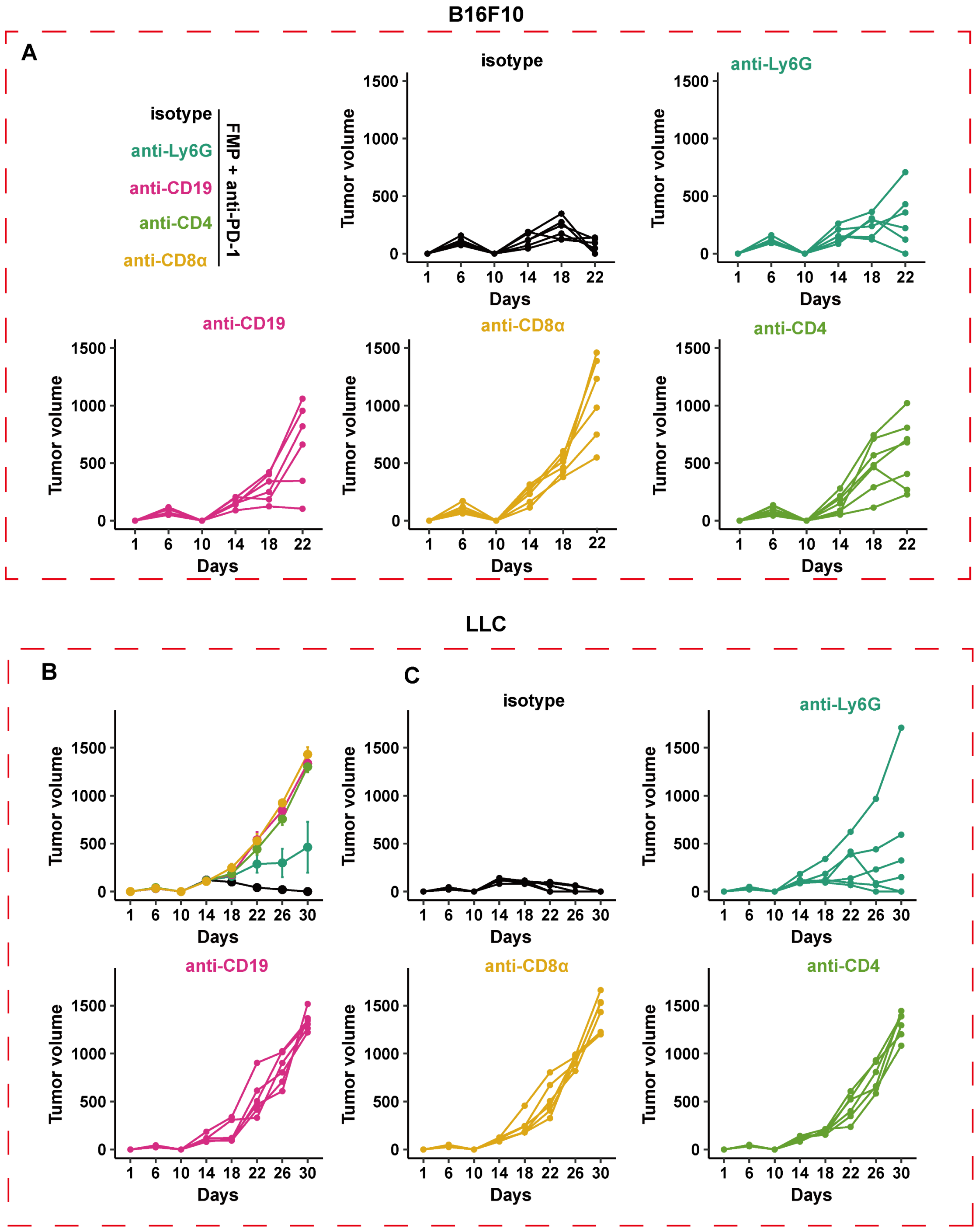

Supplementary Figure 10. Both innate and adaptive immunity are required for tumor recurrence after personalized Bio-HCP@FM-NPs vaccination, related to Figure 7. (A) B16F10 tumor-growth curves for each mouse in different groups (n = 6). (B) Average tumor-growth curves in LLC-bearing mice pre-treated with different monoclonal antibodies, before the combination treatment of Bio-HCP-NPs vaccination

and anti-PD-1 (n = 6). (C) LLC tumor-growth curves for each mouse in different groups (n = 6).

Table S1. Elemental analysis of XPS in series polymer nanoparticles.

**Sample**	**C (Atom %)**	**O (Atom %)**
**PS**	82.16	17.84
**PS@PEG-NPs**	79.02	20.98
**Bio-HCP-NPs**	91.58	8.42

Table S2. Specific surface area (SBET), average pore size (DBJH) and pore volume ((Vp)) of series Bio-HCP-NPs before and after loaded drug.

**Sample**	S_{BET} **(m²/g)**	D_{BJH} **(nm)**	V_P **(cm⁻³/g)**
**Bio-HCP-NPs**	8.08	24.45	0.06
**PS@PEG-NPs**	907.05	6.65	1.02
**Bio-HCP-NPs@GM-CSF**	349.33	7.50	0.57

Table S3. Specific surface area (SBET), average pore size (DBJH) and pore volume ((Vp)) of series Bio-HCP-NPs formed from different proportions of monomers.

**Sample**	S_{BET} **(m²/g)**	D_{BJH} **(nm)**	V_P **(cm⁻³/g)**
**90/10 St/EG**	907.05	6.65	1.02
**80/20 St/EG**	873.29	3.73	0.55
**70/30 St/EG**	624.26	6.31	0.40

**60/40 St/EG**	406.74	11.96	0.78
